# Effects of household and neighbourhood attributes on four definitions of multimorbidity: a comparative multilevel analysis of linked clinical and census data of Wales

**DOI:** 10.1136/bmjph-2025-002878

**Published:** 2026-07-20

**Authors:** Eleojo Oluwaseun Abubakar, Clare MacRae, Chunyu Zheng, Laurence Rowley-Abel, Bruce Guthrie, Chris Dibben, Jamie Pearce, Alan Marshall

**Affiliations:** 1 Department of Public Health, Policy and Systems, University of Liverpool, Liverpool, UK; 2 Usher Institute of Population Health Sciences and Informatics, The University of Edinburgh, Edinburgh, UK; 3 School of GeoSciences, The University of Edinburgh, Edinburgh, UK; 4 Social Policy, The University of Edinburgh, Edinburgh, UK; 5 Advanced Care Research Centre, University of Edinburgh, Edinburgh, UK

**Keywords:** Public Health, Demography, Epidemiology

## Abstract

**Introduction:**

To compare the influence of household and neighbourhood factors on the risk of four definitions of multimorbidity (MM), namely (1) patients with two or more long-term conditions (LTCs) (2+MM), (2) patients with three or more LTCs and (3) patients with three or more LTCs from three or more International Classification of Diseases (10th Revision) body systems and (4) 2+MM patients with at least one mental LTC and one physical LTC.

**Methods:**

We used individual-level census data of Wales and health episode records from the Secured Anonymised Information Linkage databank. The cohort was all living people registered with a Welsh general practice on the 2011 census date (27 March) (n=1 676 432). Three-level multilevel logistic regression models with individuals in households in neighbourhoods were applied to each MM definition.

**Results:**

Markers of socioeconomic disadvantage at the household (eg, tenure of dwelling) and neighbourhood (eg, neighbourhood deprivation) levels were associated with higher risks of MM across all definitions. Relative to urban cities and towns, rural village settings were associated with lower odds of all MM definitions in the fully adjusted models. Our partitioning of the variance in MM risk into level-wise components revealed more variance in households (between 23% and 12%) than in neighbourhoods (approximately 3%) and a sensitivity of the results to the definition of MM used. Household attributes accounted for a greater proportion of R^2^ in mental–physical MM (23%) than in the other definitions (8.5% to 9.5%), which is in line with research that has demonstrated a ‘social contagion’ of mental health conditions within households.

**Conclusions:**

Pertinent household and neighbourhood factors should be prioritised for targeted public interventions, and optimal strategies might vary according to definitions of MM employed, with mental-physical MM showing a different pattern from the other definitions, which have a similar pattern.

**WHAT IS ALREADY KNOWN ON THIS TOPIC**

- Multimorbidity is a key global challenge for health and social care systems, which is associated with greater use of health services, adverse healthcare events and poorer health outcomes.
- Little is known about how contextual factors in households and neighbourhoods might influence the risk of different measures of multimorbidity.

**WHAT THIS STUDY ADDS**

- Regardless of how multimorbidity is defined, markers of socioeconomic disadvantage at the household and neighbourhood levels are associated with increased risk of multimorbidity.
- The influence of the household context was substantial whereas the neighbourhood context showed a more modest influence.
- Contextual effects appear sensitive to the definition of multimorbidity employed and the control (individual-level) variables included in the models, with mental–physical multimorbidity showing a marked deviation from the other definitions.

**HOW THIS STUDY MIGHT AFFECT RESEARCH, PRACTICE OR POLICY**

- Future research and policy will benefit from separating mental–physical multimorbidity from other multimorbidity definitions.
- For mental–physical multimorbidity, household factors should be given greater consideration due to a tendency for intrahousehold social contagion of mental health conditions.

## Introduction

 Recent improvements in longevity alongside population ageing have resulted in an increase in the number of people experiencing multimorbidity (MM).^1 2^ The proportion of MM patients in the UK is projected to increase from 54% in 2015 to 68% in 2035.^3^ The deleterious effects of MM are well documented, including reductions in quality of life and life satisfaction, increased healthcare costs and excess mortality.^4^ Effective management of patients with MM requires complex and costly healthcare support across primary and secondary care providers.^5^ Polypharmacy, which has many adverse consequences, including hospital readmission, negative drug events and mortality, is also largely driven by MM.^6^,^8^

Social inequalities in MM are stark; for example, the onset of MM occurs 7 years earlier in the most deprived parts of the UK than in the most affluent.^9^ Like single acute or chronic health conditions, social disparities in MM are thought to be shaped by a plethora of demographic, socioeconomic, environmental and commercial markers that can operate at different ‘levels’, including the individual (such as personal experience of unemployment), household (such as tenure) and neighbourhood (such as deprivation or urban vs rural).^10^,^14^ Lifecourse frameworks highlight how early disadvantage, low education, unemployment and chronic stress accumulate to increase later MM risk.^10 11^ At the household scale, material constraints such as rental tenure, overcrowding or limited transport and employment resources exacerbate shared stress, caregiving strain and health deterioration.^12 13^ At the neighbourhood level, area deprivation, poor environmental quality and urban–rural contrasts shape exposure to stressors and access to supportive infrastructure.^14^ These studies confirm that multilevel determinants jointly structure both the prevalence and clustering patterns of chronic conditions, underscoring MM as a socially patterned phenomenon driven by cumulative socioeconomic, environmental and contextual disadvantage. In this study, neighbourhoods refer to small geographic areas, known as Lower Super Output Areas (LSOA) in the UK, which contain between 400 and 1200 households, comprising a residential population of between 1000 and 3000 people.^15^ Correspondingly, a household (in the UK) is a housekeeping unit composed of either one person living alone or a group of people, not necessarily related, living in the same shared house address.

Interest in multiple definitions of MM has produced substantial heterogeneity in its measurement and, consequently, in the identification of its risk factors. As systematic reviews demonstrate, studies differ in the number and type of long-term conditions (LTCs) included, the data sources used and whether conditions are weighted or grouped by body system, leading to inconsistent findings for predictors such as rural–urban residence, household composition and housing tenure.^11 13 14 16 17^ Some studies find higher MM prevalence in urban areas, attributing this to pollution, deprivation and lifestyle risks,^11 13^ whereas others show greater MM in rural regions after adjusting for age, reflecting older population structures and limited healthcare access.^14^ Still others report no significant rural–urban differences once socioeconomic composition is controlled, underscoring sensitivity to model specification and MM definition.^17^ Findings vary from evidence that living alone or in single-person households increases MM risk—via isolation and limited social support^16^—to results showing higher MM among multiperson households, possibly due to shared stress exposures, caregiving strain or socioeconomic clustering.^13^ These opposite directions often reflect whether analyses adjust for age, gender and deprivation or the inclusion of mental health conditions in MM counts. While some studies link rented accommodation to higher MM through material deprivation and residential instability,^11 16^ others find weak or non-significant tenure effects once occupational and educational status are included,^17^ and a few report protective associations of home ownership only for certain MM clusters such as cardiometabolic or mental–physical combinations.^13^ Together, these examples demonstrate how divergent definitions of MM, measurement of contextual variables and adjustment strategies produce inconsistent findings across studies—reinforcing the need for harmonised, multidimensional conceptualisations of MM. These definitional differences complicate comparisons across populations and impede synthesis of evidence on the social determinants of MM. Recent scholarship therefore urges moving beyond the simple ‘two or more LTCs’ criterion to incorporate multiple operationalisations—for example, counts based on body-system diversity or combinations of mental and physical conditions—to capture the heterogeneity and clinical relevance of disease clustering.^18^,^20^ Such multidimensional approaches better reflect the complex ways MM manifests across demographic and socioeconomic groups, enabling more consistent evaluation of its social and contextual drivers.

Studies that explore variation in health outcomes at different levels tend to use multilevel models to explore the social determinants of health outcomes across individual levels and their broader contextual environments. The focus of such models is often on individual and neighbourhood levels, without including households as a level, partly because many social surveys collect a full set of information from one member of a household (eg, Godhwani *et al*^21^ Marshall *et al*^22^ Craig^23^). The flaw of missing a salient ‘level’ in a multilevel model is well known and has been shown to lead to errors where variation in the outcome is apportioned to the other levels that are included in the model.^24^ In line with this observation, research on mental health that has included levels of individuals, households and neighbourhoods within models (eg, Weich *et al*^25^ Fone *et al*^26^) has revealed a smaller influence of neighbourhoods than studies that do not include the household level.^22 23^ A systematic review^16^ confirmed that most studies exploring the social determinants of MM either focus only on personal attributes or neglect attributes of people’s households when other intervening contexts are considered, resulting in a partial understanding of MM risk.

Documented risk factors may be sensitive to the definition of MM adopted, the ‘levels’ that are included in models (eg, individual, household and neighbourhood) and the characteristics of covariates employed across empirical studies. Policies to reduce MM inequalities require robust evidence on the relative influence of individual, household and neighbourhood risk factors on specific forms of MM. To address these research and policy needs, we used a large cohort derived from the linkage of Welsh health episode records (HERs) with the population census and other administrative data. This study aims to enrich our understanding of the risk factors of four definitions of MM through a comparative multilevel analysis focusing on the relative influence of household and neighbourhood factors. The research questions are as follows: (1) Does the relative importance of household and neighbourhood characteristics in determining multimorbidity risk vary across multimorbidity definitions? (2) In what ways do the household and neighbourhood contexts explain different multimorbidity definitions?

## Materials and methods

### Data types and sources

The key sources of data for this study were the HER, census individual-level data, and neighbourhood attributes. The HER comprises two main data sources: (1) data from general practitioner (GP) practices, known as the Welsh Longitudinal General Practice dataset and (2) hospital admission records, known as Patient Episode Data for Wales. These data were pseudonymised and provisioned by the Secure Anonymised Information Linkage (SAIL) Databank of Wales. Since there is no universally agreed-upon list of LTCs for characterising MM, we adopted the 47 LTCs used in other recent studies based on the SAIL database.^27^ Like Nguyen *et al*^28^ and Bendayan *et al*,^29^ we binary-coded the outcome variables according to four definitions of MM, namely (1) patients with two or more LTCs (2+MM), (2) patients with three or more LTCs (3+MM), (3) patients with three or more LTCs from three or more International Classification of Diseases, 10th Revision body systems (‘3+from 3+’ MM) and (4) 2+MM patients with at least one mental LTC and one physical LTC (mental-physical MM).^18 19^ Other interlinked datasets provided through the SAIL were person-level and household-level variables selected from the 2011 census, which are outlined in table 1. Because the 2011 census data were the most recent data available at the time of this study, we used neighbourhood data from around this period. This included the 2011 rural–urban classification of neighbourhoods obtained from the Office for National Statistics and the Welsh Index of Multiple Deprivation of 2011 obtained from the government’s official website.

**Table 1:** Cohort table containing key descriptive statistics of the study population

Characteristic	N=1 676 432
Response variables: four definitions/measures of MM	
2+MM	574 410 (34.26%)
3+MM	364 684 (21.75%)
‘3+from 3+’ MM	283 542 (16.91%)
Mental-physical MM	188 281 (11.23%)
Level 3 variables: neighbourhood characteristics	
ONS 2011 Rural‒Urban class	
Urban city and town	1 174 156 (70.04%)
Rural town and fringe	281 413 (16.79%)
Rural village and dispersed	220 863 (13.17%)
WIMD2011 quintile	
Least	352 871 (21.05%)
4	314 905 (18.78%)
3	349 530 (20.85%)
2	331 528 (19.78%)
Most	327 598 (19.54%)
Level 2 variables: household characteristics	
No. of cars/vans in household	
None	255 224 (15.22%)
1	656 098 (39.14%)
2 or more	765 110 (45.64%)
Family status	
Couple family	1 176 341 (70.17%)
Lone parent family	190 649 (11.37%)
Students, short-term migrants and others not in a family	309 442 (18.46%)
Household size	
1	249 379 (14.88%)
2	600 533 (35.82%)
3	344 367 (20.54%)
4 or more	482 153 (28.76%)
No. of adults in employment in household	
1	495 688 (29.57%)
2	414 008 (24.70%)
3 or more	766 736 (45.74%)
Accommodation type	
Detached whole house or bungalow	502 242 (29.96%)
Semidetached whole house or bungalow	575 139 (34.31%)
Terraced whole house or bungalow	471 550 (28.13%)
Flat, maisonette or apartment	124 166 (7.41%)
Mobile or temporary structure	3335 (0.20%)
Tenure of dwelling	
Owned or shared ownership	1 251 080 (74.63%)
Private rented	176 322 (10.52%)
Social rented	230 590 (13.75%)
Living rent free	18 440 (1.10%)
NS-SEC of household Reference Person	
Higher managerial, administrative and professional occupations	543 954 (32.45%)
Intermediate occupations	176 997 (10.56%)
Lower supervisory and technical occupations	179 655 (10.72%)
Never worked and long-term unemployed	77 625 (4.63%)
Routine and semiroutine occupations	492 368 (29.37%)
Small employers and own account workers	205 833 (12.28%)
Central heating	
No central heating	29 084 (1.73%)
Has central heating	1 647 348 (98.27%)
Level 1 variables: person characteristics	
Age grouping (10 years)	
16–24	211 181 (12.60%)
25–34	230 165 (13.73%)
35–44	263 961 (15.75%)
45–54	294 130 (17.55%)
55–64	275 058 (16.41%)
65–74	220 471 (13.15%)
75–84	131 031 (7.82%)
85 and over	50 435 (3.01%)
Sex	
Female	883 943 (52.73%)
Male	792 489 (47.27%)
Ethnic group	
White	1 623 703 (96.85%)
Mixed/multiple ethnic groups	11 690 (0.70%)
Asian/Asian British	29 197 (1.74%)
Black/African/Caribbean/Black British	6838 (0.41%)
Other ethnic groups	5004 (0.30%)
Provision of unpaid care	
Does not provide care	1 417 311 (84.54%)
Provides care	259 121 (15.46%)
Highest level of qualification	
Level 4	413 208 (24.65%)
Level 3	185 779 (11.08%)
Apprenticeship	68 910 (4.11%)
Level 2	266 447 (15.89%)
Level 1	225 038 (13.42%)
Students	19 961 (1.19%)
No qualifications	428 830 (25.58%)
Others	68 259 (4.07%)

MM, multimorbidity; NS-SEC, National Statistics Socio-economic Classification; ONS, Office for National Statistics; WIMD, Welsh Index of Multiple Deprivation.

The 2011 population of Wales was 3.1 million, comprising 1.3 million households spread across the 1909 LSOAs.^30^ However, the cohort for this study included only people aged 16 years and above whose GP practices were registered with SAIL on 27 March 2011 and who had complete records for all relevant covariates. This resulted in a cohort size of 1 676 432 persons (in 948 266 households across 1889 neighbourhoods), as described in table 1. Online supplemental material 1 (the cohort building process) provides further details on the cohort-building process, while a description of non-standard variables is contained in online supplemental material 2 (description of categories in non-standard variables).

### Methods of data analysis

The cohort had a hierarchical grouping structure of people nested in households nested in neighbourhoods (LSOAs), as shown in table 1. Therefore, the data is well suited for a nested three-level multilevel logistic model (MLM) design with persons, households and neighbourhoods belonging to the first, second and third levels, respectively. With this, we fitted random intercept models to elicit disparities in MM risk between individuals, households and neighbourhoods. The variables were entered into the models in level-wise blocks, with a view to distilling neighbourhood and household effects on MM risk. Thus, four MLM specifications were fitted on each outcome as follows:

1. Model 1: variance component (no variables).
2. Model 2: neighbourhood variables.
3. Model 3: neighbourhood and household variables.
4. Model 4: neighbourhood, household and person attributes.

Laplace transformation was used to estimate the MLMs based on the restricted maximum likelihood. This was implemented via the glmmTMB package, which is one of the most robust packages in R for fitting MLMs on large datasets.^31^ To understand the contributions of each level to the variance in MM risk, the R^2^ values of the fully adjusted models (model 4) were decomposed into level-wise components. Vital information criterion metrics,^32^ which serve as additional goodness-of-fit measures, were also computed. Furthermore, we estimated the variance partition coefficient (VPC) (equivalent to the intraclass correlation coefficient (ICC) in a random intercept model) for each model and partitioned the variance into individual, household and neighbourhood components.^33^

In addition to explicating the effects of covariates on the outcomes of interest, MLMs are designed to properly handle dependencies when analysing data whose variables are clustered at different levels, such as the household and neighbourhood contexts of the present study. While other advantages of MLM are detailed in Chimwanda *et al*^34^ and Griffith and Jones,^35^ for this study, they are crucial for answering aspects of the research questions, such as (1) unpicking the relative importance of the levels to MM risk across various definitions and (2) uncovering within group homogeneity (clustering) of MM risk in households and neighbourhoods through ICCs. MLMs also provide the benefit of revealing neighbourhoods with higher-than-expected risk of MM, which could constitute potential targets for relevant public health interventions and increased resource allocation.

### Patient and public involvement

No patient involved.

## Results

### Effects of neighbourhood and household factors on MM risk profiles

**Figure 1:**
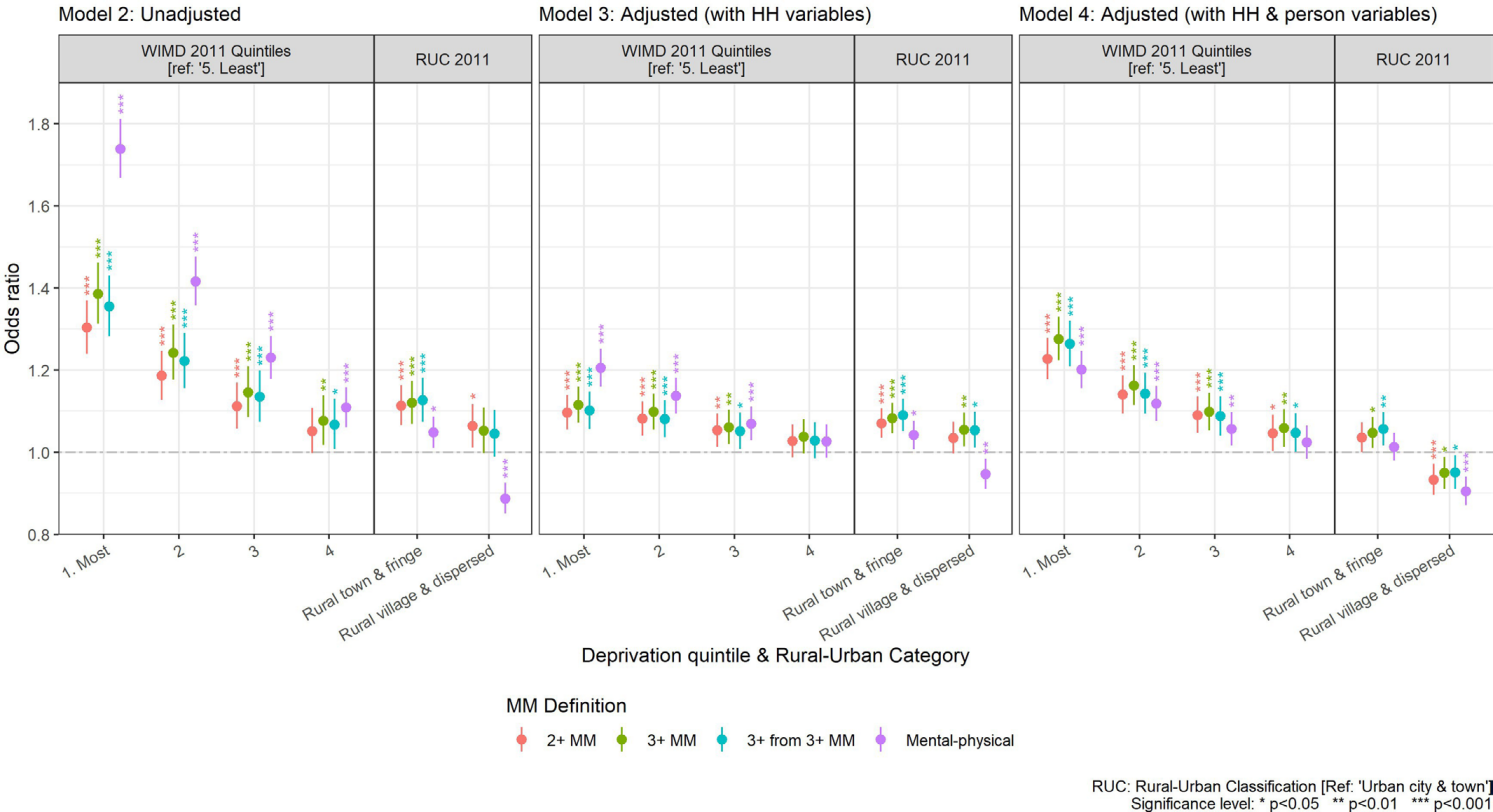
Pre-adjustment and post-adjustment effects of neighbourhood factors on the four definitions of multimorbidity. HH, household; MM, multimorbidity; RUC, rural–urban classification; WIMD, Welsh Index of Multiple Deprivation.

The relative risk (of the four definitions) of MM across the two neighbourhood variables considered in this study is depicted in figure 1, which also portrays the effects of adjusting for household-level (model 3) and then person-level attributes (model 4). Across all the models, we observe a social gradient of decreasing MM risk with decreasing neighbourhood deprivation, which is most pronounced with mental–physical MM, prior to adjusting for household and personal factors.

We found that the urban–rural character of neighbourhoods had an association with MM that is sensitive to the definition of MM and the control variables that are included in the model. In the fully adjusted model (model 4), we observed lower odds of MM in ‘rural village and dispersed’ settings than in ‘urban city and town’ settings for all our definitions of MM. For example, the odds of mental–physical MM in the fully adjusted model were 10% lower (OR 0.9, 95% CI 0.87 to 1.05) in neighbourhoods classified as ‘rural villages and dispersed’ than in those classified as ‘urban city and town’. While this direction of association held across all models for mental–physical MM, including the unadjusted model, this was not the case for the three other definitions of MM that we explored (2+MM, 3+MM and ‘3+from 3+’ MM). For these measures of MM, we found the opposite association in the unadjusted (model 2) and partially adjusted models (model 3), with higher odds of MM in ‘rural village and dispersed’ settings than in ‘urban city and town’ settings, as shown in figure 1.

**Figure 2:**
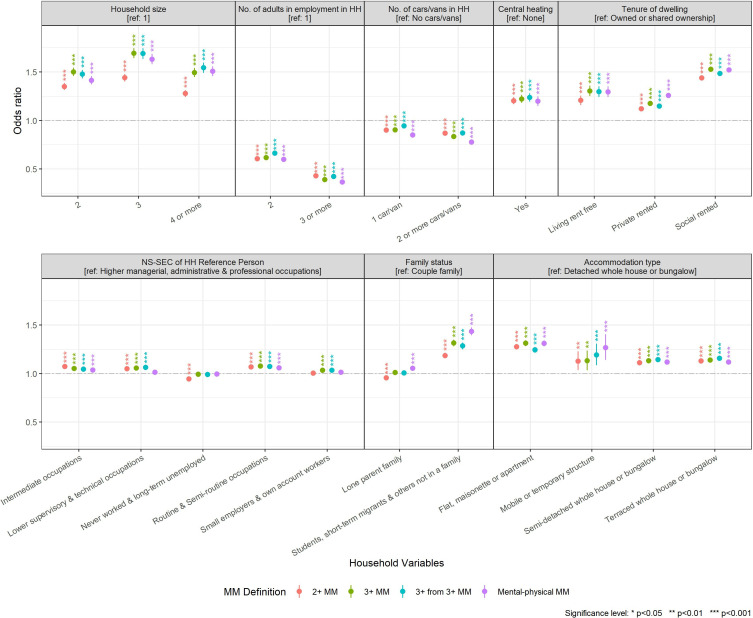
Effects of household attributes on the four definitions of multimorbidity after full adjustments (model 4). HH, household; MM, multimorbidity; NS-SEC, National Statistics Socio-economic Classification.

Figure 2 shows that the association between household variables and MM is not sensitive to how MM is defined and is broadly in line with theories on the social determinants of health. For example, for all definitions, we find higher odds of MM among those who rent their home; who live in households with 2, 3 or 4+ people; who live in non-detached accommodation (eg, a flat or terrace); whose household reference person is involved in routine work; and who live in multi-member households that are not connected by family ties (OR 1.19, 95% CI 1.16 to 1.21 for 2+ MM; OR 1.31, 95% CI 1.28 to 1.35 for 3+ MM; OR 1.28, 95% CI 1.25 to 1.32 for 3+ from 3+ MM and OR 1.43, 95% CI 1.39 to 1.48 for mental–physical MM). We find lower odds of all forms of MM for those households with 2 or 3+ adults in employment and who have access to one or more cars/vans. Online supplemental material 3 (ORs and random effects of the models) provides the coefficients of the fixed and random effects for all the models (see online supplemental Tables SM3-1 to SM3-4).

### Homogeneity of MM risk within neighbourhoods and households

We use our random intercept multilevel models to calculate the VPCs, which provide a useful summary of the percentage of the variation in log odds of MM that is attributable to the household and neighbourhood levels of our models. These are presented in table 2 (and online supplemental figure SM4-1), from which we derive three key findings. First, our results show that households have a significant influence on MM, with VPC varying between 21.6% and 11.9% in the variance component model across our definitions of MM. Given the well-known risk of missing a level in a multilevel model,^24^ we can be more strongly assured in the robustness of our estimates of approximately 3.0%–3.5% of the variation in the log odds of MM being at the neighbourhood level in our variance component model.

**Table 2:** R^2^ and ICC values of the models (with level-wise decomposition)

Definition of MM	Model specification	Marginal R^2^	Conditional R^2^	Total ICC	Household ICC	Neighbourhood ICC
2+MM	Model 1	0.0%	22.4%	22.4%	19.4%	3.0%
2+MM	Model 2	0.2%	22.4%	22.2%	19.5%	2.7%
2+MM	Model 3	24.7%	30.8%	8.1%	6.4%	1.7%
2+MM	Model 4	41.6%	45.5%	6.6%	4.6%	2.0%
3+MM	Model 1	0.0%	25.0%	25.0%	21.6%	3.3%
3+MM	Model 2	0.3%	24.9%	24.7%	21.7%	3.0%
3+MM	Model 3	28.4%	34.7%	8.8%	7.0%	1.8%
3+MM	Model 4	48.7%	52.4%	7.2%	5.2%	2.0%
3+LTC from 3+body systems	Model 1	0.0%	23.3%	23.3%	19.9%	3.5%
3+LTC from 3+body systems	Model 2	0.3%	23.3%	23.1%	19.9%	3.1%
3+LTC from 3+body systems	Model 3	27.5%	33.9%	8.7%	6.8%	2.0%
3+LTC from 3+body systems	Model 4	50.5%	54.0%	7.1%	4.9%	2.2%
Mental-physical MM	Model 1	0.0%	15.1%	15.1%	11.9%	3.2%
Mental-physical MM	Model 2	1.1%	14.8%	13.9%	12.1%	1.8%
Mental-physical MM	Model 3	12.9%	20.4%	8.6%	7.0%	1.5%
Mental-physical MM	Model 4	25.2%	32.3%	9.4%	7.9%	1.5%

ICC, intraclass correlation coefficient; LTC, long-term condition; MM, multimorbidity.

Second, considering the household level, the results of the VPC across the models appear to be sensitive to the definition of MM. In the variance component model, there is less clustering of MM within households for mental–physical MM than for other MM definitions. In this model, the household level accounts for approximately 20% of the variation in the log odds of MM for the 2+, 3+ and ‘3+from 3+’ definitions compared with approximately 11.9% for mental–physical MM. However, we were more successful in explaining the household clustering in MM for the 2+, 3+and ‘3+from 3+’ definitions, which decreased sharply from 19.4%, 21.6%, and 19.9%, respectively, in the variance component models to 4.6%, 5.2% and 4.9%, respectively, in the fully adjusted models. For mental–physical MM, the variation in the log odds of MM declined to a lesser extent from 11.9% in the variance component model to 7.9% in the fully adjusted model.

Third, when considering VPC at the neighbourhood level, we find the opposite result across models, with the suggestion that our models are more successful in explaining the clustering of MM within neighbourhoods for mental–physical MM than the other MM definitions. The neighbourhood VPC was 3.0%, 3.3% and 3.5% for the 2+, 3+and ‘3+from 3+’ definitions, respectively, in the variance component model, which reduced to 2.0%, 2.0% and 2.2%, respectively, in the fully adjusted model. For mental-physical MM, the VPC declined to a greater extent from 3.2% in the variance component model to 1.5% in the fully adjusted model. Further details on the decomposed VPC (or ICC) values from the models are provided in table 2 and online supplemental material 4 (level-wise decomposition of the ICC and R^2^ values of the models).

### Exploring the R^2^ values across MM definitions

**Figure 3:**
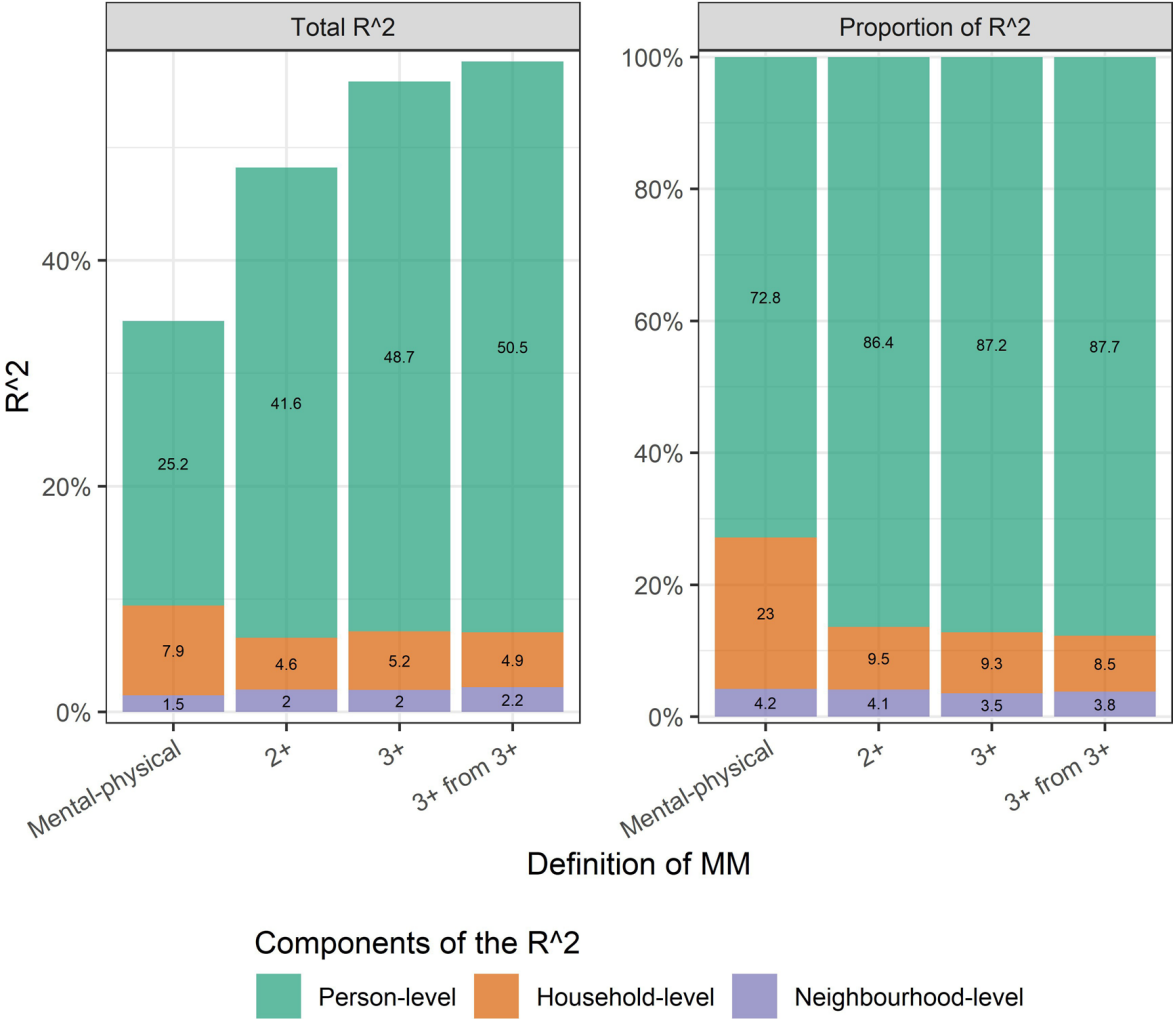
Variance explained by the fully adjusted model (model 4) decomposed according to their level-wise components. MM, multimorbidity.

The amount of variance explained by regression models is denoted by R^2^ values, which range from 0 to 1 (or 100%), with higher R^2^ values indicating greater variance explained and better goodness-of-fit. In the final set of results, we examine the R^2^ statistics in our fully adjusted model, decomposing the contribution to the R^2^ value to the blocks of individual, household and neighbourhood variables in the models. Figure 3 indicates that there is variation in the ability of our full model to explain MM across different definitions. The lowest goodness-of-fit (32.3%) was recorded for mental–physical MM, suggesting that our models are less successful with this definition than with the other definitions of MM, which achieved R^2^ values of approximately 50%, with ‘3+from 3+’ MM best explained (R^2^ of 54.0%).

Household attributes account for a greater proportion of R^2^ in mental–physical MM than in the other definitions. Given that these factors are more sensitive to both neighbourhood and household attributes, they are more important to mental–physical MM (23%) than to the other definitions (8.5%–9.5%) in this study. Therefore, unlike the other definitions, mental–physical MM appears to be more strongly impacted by an interplay of person-level and household-level factors. Further details on the decomposed R^2^ values from the models are provided in table 2 and online supplemental material 4 (level-wise decomposition of the ICC and R^2^ values of the models).

## Discussion

### Summary of findings

This paper makes four contributions to the literature. First, we show that household and area measures of socioeconomic circumstances have similar associations with MM regardless of the definition of MM that is used. Second, the effects of living in a rural village on MM are sensitive to the definition of MM and the socioeconomic and demographic control variables included in the model. Importantly, living in rural village neighbourhoods appears to be associated with lower risks of all MM definitions after controlling for compositional differences in social, economic and demographic factors. Third, we use multilevel models that include households and neighbourhoods as levels to more accurately estimate the percentage of variation in MM risk at each level compared with models that do not apply a multilevel design or that include only neighbourhood and individual levels. Fourth, we offer evidence that the importance of the household and neighbourhood levels in determining MM risk varies according to the MM definition, as does our understanding of the socioeconomic factors that underpin the clustering at each level.

### MM and multilevel socioeconomic disadvantage

Markers of socioeconomic disadvantage at the household and neighbourhood levels are associated with increased risks of MM for all definitions of MM. We observed similar effect sizes and little evidence of any systematic differences in these associations across the four MM definitions. This finding is in line with the literature on multilevel social determinants of MM, whereby the experience of social and economic disadvantage at various levels leads to poorer outcomes in various definitions of MM, including those that count both physical and mental LTCs.^9 14 16 28 29 36 37^ The indicators of socioeconomic disadvantage considered in the present study include a lack of car ownership, living in relatively disadvantaged neighbourhoods, a lack of home ownership, and living in a flat, maisonette or apartment. This gradient is expected, given well-established SDH theories that explain mechanisms linking multiple facets of deprivation with poor health outcomes, motivating some scholars to propose socioeconomic status (SES) as a fundamental cause of mortality.^38^ However, explanations of health determinants based exclusively on SES can fall short in occasions where pertinent attributes are not socially gradational or where it is not apparent which categories symbolise better socioeconomic circumstances that are also health-promoting (such as household size and rural–urban classification). For example, although single-person households are often linked with relatively poor well-being and/or socioeconomic disadvantage,^39^ in the present study, multi-resident households presented greater MM risk across all definitions, but this contradicts patterns observed in other studies.^16^ On the one hand, this can indicate that it is not living alone that is deleterious to health, but other attributes frequently associated or conflated with single-person households, such as their increased risk of loneliness or social isolation. On the other hand, alternative frameworks for explaining the potential effects of social circumstances on MM risk are necessary complements of those based solely on SES.

Among the many models, frameworks and theories provided by the SDH literature for understanding the multiple pathways linking markers of social status with health outcomes, biopsychosocial models and psychological stress processes stand out.^40^,^42^ This is especially true for determinants of MM, which is highly associated with geriatrics, not least because several studies indicate that chronic stress is intertwined with ageing, LTCs and mortality via the intermediary of allostasis and allostatic (over)load.^43^,^47^ According to the stress process model,^48 49^ a key pathway by which many facets of socioeconomic circumstances can increase MM risk and, by extension, result in poor health outcomes is the increased and sustained stress experienced by people of disadvantaged SES alongside their limited resources to cope or adapt.^37 50 51^ For example, in the present study, people who lack a car, have a larger household size or are in strenuous occupational categories are likely to experience greater stress than others; thus, they exhibit a relatively greater risk of MM. Difficulties in meeting key (financial) needs and/or being in precarious or less favourable circumstances are also major psychological stressors associated with socioeconomic disadvantage. The stress-mediated psychological effects of social disadvantage often manifest as higher allostatic loads (AL) and greater premature ageing (weathering), which are deleterious to health, thereby increasing the risk of MM.^4045 47 48 51^,^53^ This is an alternative explanation for the association between pertinent facets of SES and MM risk observed in the present study, reverberating the specialised literature.

### MM in urban and rural settings

We extend the literature on neighbourhood effects on MM (such as Head *et al*^9^ Knies and Kumari^36^) to move beyond neighbourhood deprivation to consider urban/rural classifications as another feature of neighbourhoods. We found lower risks of physical–mental MM in ‘rural village and dispersed’ settings across all models, and for other definitions of MM in the fully adjusted model. In contrast, living in a ‘rural village and dispersed’ neighbourhood was associated with higher levels of MM defined by 2+, 3+ and ‘3+from 3+’ measures in unadjusted or partially adjusted models. The inconsistent results here are likely driven by age differences between rural and urban areas and the rise in mental health conditions in middle-aged and younger adults, which makes the prevalence of mental–physical MM less concentrated at older ages.^54^ Rural areas tend to be more elderly than urban areas, and the rates of MM are particularly concentrated at older ages for 2+, 3+ and ‘3+from 3+’ definitions of MM. Thus, the unadjusted and partially adjusted models revealed elevated risks of these three definitions of MM in rural areas. Research has also revealed that health-promoting aspects of neighbourhood character, such as access to green space and high levels of community cohesion, are more prevalent in rural areas than in urban areas.^55^ Such differences between urban and rural settings may well explain the lower risk of all types of MM in ‘rural village and dispersed’ areas after controlling for compositional and contextual differences in socioeconomic deprivation.

### MM risk and the relative influence of individual, household and neighbourhood levels

Our analysis to partition the variance in MM across individual, household and neighbourhood levels reveals a significant influence of individual and household levels and a more modest influence of the neighbourhood level. We are not aware of such an analysis being undertaken to explore the variation in MM across individual, household and neighbourhood levels. Our findings are broadly consistent with the literature on mental health outcomes, which also report relatively smaller neighbourhood-level effects for mental health outcomes when households are included as a level in the model.^35 56^ While we found that neighbourhoods account for between 3.5% and 1.5% of the variance across models and definitions of MM, it is important to recognise that a modest area health effect is nonetheless significant in scale where it applies across a population living in an area. Similarly, while neighbourhood health effects may be small compared with the impact of individual and household factors, markers of individual/household disadvantage are known to cluster in particular neighbourhoods, meaning that neighbourhood-focused interventions may bring advantages of efficiency in tackling health inequalities. Consequently, one conclusion from this study for MM research and policy is to reiterate the relevance of place and argue for a greater consideration of household factors in empirical studies and in policy interventions.^16 57^

### Neighbourhood and household influence on MM: sensitivity to MM definition

Our findings concerning the importance of household and neighbourhood levels through the VPCs appear sensitive to the definition of MM as well as the independent variables included in the model. In the unadjusted (variance components) model, households are more influential for 2+, 3+and ‘3+from 3+’ MM definitions than for mental–physical MM definitions. However, the reverse is true in the fully adjusted model that controls for individual, household and neighbourhood variables, where households are more influential for mental–physical MM than for the other three definitions of MM. It seems then that clustering of our control variables within households explains the greater importance of households in determining variation in MM defined according to the 2+, 3+ and ‘3+from 3+’ measures. The fully adjusted models indicate that households are more important in influencing variation in mental–physical MM for reasons that are not included in our model. Similarly, independent variables at the household level contribute the most to model fit for physical‒mental MM, indicating the importance of household circumstances for MM risk once mental health issues are given greater priority. These results are consistent with research that has revealed a ‘social contagion’ of mental health issues within households, which might explain the greater importance of households for mental–physical MM than the other MM definitions since we do not control for this in our models.^58 59^ In a broader sense, the sensitivity of our results on the VPC across MM definitions supports the recommendation to consider multiple definitions of MM.^14 18^ In a policy sense, initiatives concerned with ameliorating mental–physical MM would also benefit from increased attention to household conditions.

### Implications for research, social policy and practice

The study’s findings have important implications for public-health policy and service design. The persistent association between socioeconomic disadvantage and MM across both household and neighbourhood contexts underscores the need for place-sensitive and household-sensitive interventions rather than exclusively person-centred approaches. Policies should be guided by the principle of proportionate universalism—universal interventions delivered with intensity according to need—so that deprived households and neighbourhoods receive additional support without fragmenting population coverage.^60 61^ In practice, this involves integrating public-health and primary-care initiatives with local-authority housing, social-care and welfare services to target households in social-rented or multi-occupancy dwellings and neighbourhoods characterised by deprivation, unemployment or poor transport access.

The pronounced role of the household, particularly in mental–physical MM, suggests that the household should be recognised as a key unit of policy and preventive intervention. Intra-household clustering of mental-health conditions points to potential benefits from family-based prevention programmes, social-prescribing schemes and caregiver-support interventions that address shared stress exposures and caregiving strain. Routine collection of household composition, tenure and employment data in health records could facilitate risk stratification and allow more tailored outreach. Although neighbourhood effects were smaller, they remain meaningful at population scale, reinforcing the value of area-level investments in housing quality, energy efficiency, green spaces and community cohesion. Such improvements can reduce chronic-stress pathways and environmental burdens that contribute to the accumulation of LTCs.

Finally, the observed sensitivity of contextual effects to MM definition has implications for monitoring and research. Public-health surveillance should employ multiple operationalisations—such as condition counts, body-system diversity and mental–physical combinations—to ensure equitable recognition of socially patterned forms of MM and to guide anticipatory health-service planning. More broadly, sustainable progress requires addressing the upstream determinants of MM—employment insecurity, educational inequities, fuel poverty and housing instability—through multisectoral action spanning health, housing and welfare systems. Embedding these insights within frameworks such as The Well-being of Future Generations (Wales) Act would enable cross-sectoral resource targeting, aligning with a broader strategy that situates the individual within their household and residential context. In this way, proportionate, household- and place-responsive policies can most effectively curb the population burden of MM and narrow entrenched health inequalities.

### Strengths of the study

This study makes several distinctive contributions to the literature on the social determinants of MM. Drawing on linked individual-level census data and electronic health records from the SAIL Databank, it offers an exceptionally comprehensive and population-wide examination of how demographic, socioeconomic and contextual factors interact across scales. The linkage between census and clinical data enables high-resolution modelling of MM across 1.6 million adults in Wales, overcoming common weaknesses of self-reported or limited clinical samples and ensuring robust external validity. The combination of national coverage, detailed socioeconomic information and the use of multilevel variance partitioning and level-wise R^2^ decomposition provides one of the most statistically rigorous estimates to date of contextual variation in MM risk.

A major methodological innovation of the study is the explicit inclusion of the household as an intermediate analytic level between individuals and neighbourhoods. This three-level multilevel design directly addresses the well-recognised ‘missing-level’ problem in contextual epidemiology,^24^ producing more accurate and interpretable variance estimates. The finding that household-level variation exceeds neighbourhood-level variation represents a substantive advance in understanding where MM risk clusters within populations, positioning the household as a critical yet often overlooked site of health production, social contagion and inequality.

Equally important, the study compares four alternative definitions of MM—simple condition counts, body-system diversity and mental–physical combinations—demonstrating that contextual effects are sensitive to how MM is conceptualised. This comparative design answers current calls to move beyond a single ‘two or more conditions’ definition^19^ and reveals stronger household effects for mental–physical MM, implying psychosocial transmission of mental-health burdens within households. Collectively, these innovations establish this study as one of the most comprehensive population-level analyses of MM to date, providing new empirical evidence and methodological guidance for future health-inequality research.

### Limitations and future directions

A cross-sectional study design that is popular in the literature was implemented in this study, even though it disregards the accumulation/progression of MM over the life course, which is crucial for understanding the social epidemiology of MM, which is highly connected with the ageing process.^10 62^ In our models, we included only a random intercept and did not explore random slopes. In this sense, we did not explore contextual variations in pertinent factors that could explicate disparities in the degree to which such (random) effects are salient in various neighbourhoods and households, as recently suggested by Griffith and Jones.^35^ We did not analyse the potential for intersectional drivers of MM risk with the multilevel analysis of individual heterogeneity and discriminatory accuracy methodology, which provides a framework for such analysis. While this was beyond the scope of this paper, we recognise it as a valuable direction for future research.

By focusing only on adults, MM profiles in young individuals are disregarded, which is under-researched and deserves further investigation.^28^ Since the focus of this study is not on personal attributes, we did not adjust for behavioural, psychological and lifestyle determinants of health (such as smoking, drinking, physical activity and nutrition)^28 63^ or other crucial factors, such as migration status and healthcare utilisation.^64 65^ These are potentially relevant to future studies concerned with individual characteristics. Only two neighbourhood factors were incorporated, with the implication that a limited understanding of the effects of place on MM is provided. We did not consider how the Welsh population structure of 2011 compares to their present population structure and that of other nations of the UK, which might limit the generalisability of this study. For example, alongside continued population ageing, accelerated population migration in recent times would have resulted in greater ethnic diversity. Historical and geographical dynamics in urbanisation and industrialisation are also in force, suggesting the need for future studies to confirm our findings with more recent data and for other nations of the UK or elsewhere. Although relevant to public health policy and matters of health inequality, count-based definitions of MM, such as those used in our present study, are of limited relevance to clinical practice and patients for whom more homogeneous/precise definitions of MM, such as chronicity profiles, tend to be more useful.^66^

## Conclusions

This study supports many of the widely observed patterns of associations between pertinent socioeconomic attributes and MM risk in a whole population cohort. In addition to featuring population attributes seldom considered in the literature, we show that regardless of how MM is measured or defined, its risk increases with less favourable socioeconomic circumstances at the individual, household and neighbourhood levels. We extend research that has connected area deprivation and MM to consider the rural–urban gradient and find lower risks of MM after controlling for other individual, household and area confounding variables. The rich MLM design implemented allowed us to partition the variance in the risk of MM to individual, household and neighbourhood levels, overcoming a common issue of missing the household level and, in doing so, revealing significant clustering of MM within households. We found that household clustering is particularly relevant for mental–physical MM and offer the social contagion of mental health issues within households as a possible mechanism for our empirical results.

## Supplementary material

10.1136/bmjph-2025-002878 online supplemental file 1

10.1136/bmjph-2025-002878 online supplemental file 2

10.1136/bmjph-2025-002878 online supplemental file 3

10.1136/bmjph-2025-002878 online supplemental file 4

## Data Availability

Data may be obtained from a third party and are not publicly available.
