# Supplementary material for: Effects of household and neighbourhood attributes on four definitions of multimorbidity: a comparative multilevel analysis of linked clinical and census data of Wales
Source: BMJ Public Health. 2026 Jul 20;4(3):e002878. doi: 10.1136/bmjph-2025-002878 (PMC13386087; doi:10.1136/bmjph-2025-002878)
Supplement: online supplemental file 1 [file bmjph-4-3-s001.docx]

Effects of household and neighbourhood attributes on four definitions of multimorbidity: A comparative multilevel analysis of linked clinical and census data of Wales

Supplementary Material 1

# The cohort building process

In the SAIL databank, three sets of tables were joined to produce the population cohort for this study. The first was the 2011 census tables (CENW) which provided select variables on persons and households outlined in Table SM2-1, including week of birth (WOB), sex, and tenue of dwelling and the 2011 Lower Super Output Areas (LSOA) geographies. The census records comprised 3,264,772 persons in 1,421,337 households. The second table, Wales Demographic Service Data (WDSD) provided area-based deprivation data concerning 3,092,046 persons, matching the 2011 LSOA. These included the Wales Index for Multiple Deprivation (WIMD) of 2014 and 2019 as well as the Townsend index of 2011. For each of these, the raw scores, ranks, quintiles and deciles were provided. The WDSD data was joined to the population census table such that the total number of cases in the resulting table remained the same as those in the original census table (i.e. 3,264,772) with the main difference being the addition of area-based deprivation variables. Multimorbidity data was derived from two sources of Health Episode Records (HER). HERs from hospitals were provided by the Patient Episode Data of Wales (PEDW) tables while those from GP practices were contained in the Welsh Longitudinal General Practice Dataset (WLGP) tables. These were used to derive four measures of multimorbidity for 2,472,944 SAIL-registered patients and was joined with the spatially enriched population census table via an inner join operation, such that the resulting table had 2,075,591 records. Other geographic data like the Rural-Urban data of 2011 (RUC 2011) and WIMD 2011 were obtained from the Office of National Statistics (ONS) website and joined to the population census based on pertinent LSOA codes.
