# Supplementary material for: Effects of household and neighbourhood attributes on four definitions of multimorbidity: a comparative multilevel analysis of linked clinical and census data of Wales
Source: BMJ Public Health. 2026 Jul 20;4(3):e002878. doi: 10.1136/bmjph-2025-002878 (PMC13386087; doi:10.1136/bmjph-2025-002878)
Supplement: online supplemental file 2 [file bmjph-4-3-s002.docx]

Effects of household and neighbourhood attributes on four definitions of multimorbidity: A comparative multilevel analysis of linked clinical and census data of Wales

Supplementary Material 2

# Description of categories in non-standard variables

Select variables that might not be easily understood in other study contexts (outside of the UK) are described in this section. Variables belonging in each of the levels of the multilevel models (MLM) fitted in this study are outlined in Table SM2-1.

Table SM2-1 Variables constituting the level-wise groups in the MLMs

| **Person variables**  **(Level 1): control** | **Household variables**  **(Level 2): Main** | **Neighbourhood variables (Level 3): Main** |
| --- | --- | --- |
| Age | Central heating | ONS Rural-Urban classification |
| Sex | Tenure of dwelling | WIMD 2011 |
| Ethnic group | Accommodation type |  |
| Provision of unpaid care | Family status |  |
| Education | Number of cars |  |
|  | Household size |  |
|  | Employed adults in household |  |
|  | NS-SEC of household reference person |  |

**Tenure of dwelling**

Tenure provides information about whether a household rents or owns the accommodation

that it occupies and, if rented, combines this with information about the type of landlord

who owns or manages the accommodation.

**NS-SEC of HH reference person**

The National Statistics Socio-economic Classification (NS-SeC) provides an indication of socio-economic position based on occupation. It is an Office for National Statistics standard classification. To assign a person aged 16 to 74 to an NS-SeC category, their occupation title is combined with information about their employment status, whether they are employed or self-employed, and whether they supervise other employees. Full-time students are recorded in the “full-time students” category regardless of whether they are economically active or not. Information about the classification is available: <http://www.ons.gov.uk/ons/guidemethod/classifications/current-standard-classifications/soc2010/soc2010-volume-3-ns-sec--rebased-on-soc2010--user-manual/index.html>. Within this conceptual model, the original NS-SEC classes can be collapsed into eight (8), five (5) or three (3) class versions. This study employed the 5-class re-categorisation shown in the cohort table.

**Rural-Urban classification in Wales**

The Rural-Urban Classification categorises data zones in accordance with a two-dimensional typology based on settlement form and settlement context (sparsity). Following principles set out in a review of urban and rural definitions by the Department of Communities and Local Government (DCLG) in 2006, the ‘urban’ domain comprises all physical settlements with a population of 10,000 or more. If the majority of the population of a particular Output Area (OA) live in such a settlement, that OA is deemed 'urban'; all other OAs are deemed 'rural'. Assignments of Lower Super Output Areas (LSOAs) and Middle Super Output Areas (MSOAs) to urban or rural categories are made by reference to the category to which the majority of their constituent OAs are assigned. For Wales, this results in six categories for LSOAs and MSOAs. These were recoded to three (3) categories mainly by disregarding the context dimension, as shown in Table SM2-2. Further details can be found on the government website <https://www.ons.gov.uk/methodology/geography/geographicalproducts/ruralurbanclassifications/2011ruralurbanclassification>

Table SM2-2 Rural-Urban classification and its recoding

| **Settlement form** | **Rural-urban classification** | **Recoded classes** |
| --- | --- | --- |
| Urban | City and Town | Urban city & town |
|  | City and Town in a Sparse Setting |  |
| Rural | Town and Fringe | Rural town & fringe |
|  | Town and Fringe in a Sparse Setting |  |
|  | Village and dispersed | Rural village & dispersed |
|  | Village and Dispersed in a Sparse Setting |  |

**Education**

This represents the highest educational qualification held by an individual. In the census questionnaire, 12 response options (plus ‘no qualifications’) were provided, covering professional and vocational qualifications and a range of academic qualifications. These were combined into the categories as described in Table SM2-3 below.

Table SM2-3 Educational categories of the study population

| **Category** | **Description** |
| --- | --- |
| No qualifications | No academic or professional qualifications |
| Level 1 | 1-4 O Levels/CSE/GCSEs (any grades), Entry Level, Foundation Diploma, NVQ level 1, Foundation GNVQ, Basic/Essential Skills |
| Level 2 | 5+ O Level (Passes)/CSEs (Grade 1)/GCSEs (Grades A*-C), School Certificate, 1 A Level/ 2-3 AS Levels/VCEs, Intermediate/Higher Diploma, Welsh Baccalaureate Intermediate Diploma, NVQ level 2, Intermediate GNVQ, City and Guilds Craft, BTEC First/General Diploma, RSA Diploma |
| Apprenticeship | Apprenticeship |
| Level 3 | 2+ A Levels/VCEs, 4+ AS Levels, Higher School Certificate,  Progression/Advanced Diploma, Welsh Baccalaureate Advanced Diploma, NVQ Level 3; Advanced GNVQ, City and Guilds Advanced Craft, ONC, OND, BTEC National, RSA Advanced Diploma |
| Level 4+ | Degree (for example BA, BSc), Higher Degree (for example MA, PhD, PGCE), NVQ Level 45, HNC, HND, RSA Higher Diploma, BTEC Higher level, Foundation degree (NI), Professional qualifications (for example teaching, nursing, accountancy) |
| Other | Vocational/Work-related Qualifications, Foreign Qualifications/ Qualifications gained outside the UK (NI) (Not stated/ level unknown) |
| Students | Those aged under 16 and students at their non-term-time address |
