# Supplementary material for: Effects of household and neighbourhood attributes on four definitions of multimorbidity: a comparative multilevel analysis of linked clinical and census data of Wales
Source: BMJ Public Health. 2026 Jul 20;4(3):e002878. doi: 10.1136/bmjph-2025-002878 (PMC13386087; doi:10.1136/bmjph-2025-002878)
Supplement: online supplemental file 3 [file bmjph-4-3-s003.docx]

Effects of household and neighbourhood attributes on four definitions of multimorbidity: A comparative multilevel analysis of linked clinical and census data of Wales

Supplementary Material 3

# Odds ratios and random effects of the models

For each outcome, we fitted four models: (1) Variance component, (2) Neighbourhood variables, (3) Neighbourhood and household variables, (4) Neighbourhood, household and person attributes. The odds ratios from these models are depicted in Tables SM3-1 to SM3-4. This model-building strategy was also instrumental in understanding the impact of the three categories of variables on (1) the odds ratios of the main risk factors, (2) various metrics of model fit (like the R^2^ and AIC), and (3) clustering of multimorbidity risk within households and neighbourhoods (via the level-wise intraclass correlation coefficients (ICCs)).

Table SM3-1 Fixed effects (Odds ratios) and random effects of MLMs of 2+ MM

| **Logit MLM of '2+ MLTC' in Wales: Fixed Effects for 3-Level Models** | | | | | | | | |
| --- | --- | --- | --- | --- | --- | --- | --- | --- |
|  | **Variance component** | | **Area effects** | | **Area & HH effects** | | **All Effects** | |
| *Predictors* | *Odds Ratios* | *CI* | *Odds Ratios* | *CI* | *Odds Ratios* | *CI* | *Odds Ratios* | *CI* |
| (Intercept) | 0.51 ^***^ | 0.50 – 0.52 | 0.44 ^***^ | 0.43 – 0.46 | 1.59 ^***^ | 1.52 – 1.67 | 0.04 ^***^ | 0.04 – 0.04 |
| WIMD2011 quintile: 5. Least | Reference |  | Reference |  | Reference |  | Reference |  |
| WIMD2011 quintile: 4 |  |  | 1.05 | 1.00 – 1.11 | 1.03 | 0.99 – 1.07 | 1.05 ^*^ | 1.00 – 1.09 |
| WIMD2011 quintile: 3 |  |  | 1.11 ^***^ | 1.06 – 1.17 | 1.05 ^**^ | 1.01 – 1.09 | 1.09 ^***^ | 1.05 – 1.14 |
| WIMD2011 quintile: 2 |  |  | 1.19 ^***^ | 1.13 – 1.25 | 1.08 ^***^ | 1.04 – 1.12 | 1.14 ^***^ | 1.09 – 1.19 |
| WIMD2011 quintile: 1. Most |  |  | 1.30 ^***^ | 1.24 – 1.37 | 1.10 ^***^ | 1.06 – 1.14 | 1.23 ^***^ | 1.18 – 1.28 |
| No. of cars/vans in HH: No cars/vans | Reference |  | Reference |  | Reference |  | Reference |  |
| No. of cars/vans in HH: 1 car/van |  |  |  |  | 0.79 ^***^ | 0.78 – 0.80 | 0.90 ^***^ | 0.89 – 0.91 |
| No. of cars/vans in HH: 2 or more cars/vans |  |  |  |  | 0.72 ^***^ | 0.70 – 0.73 | 0.87 ^***^ | 0.86 – 0.88 |
| Family status: Couple family | Reference |  | Reference |  | Reference |  | Reference |  |
| Family status: Lone parent family |  |  |  |  | 0.52 ^***^ | 0.52 – 0.53 | 0.96 ^***^ | 0.94 – 0.97 |
| Family status: Students, short-term migrants & others not in a family |  |  |  |  | 1.33 ^***^ | 1.30 – 1.36 | 1.19 ^***^ | 1.16 – 1.21 |
| Household size: 1 | Reference |  | Reference |  | Reference |  | Reference |  |
| Household size: 2 |  |  |  |  | 1.50 ^***^ | 1.47 – 1.54 | 1.35 ^***^ | 1.32 – 1.39 |
| Household size: 3 |  |  |  |  | 1.01 | 0.99 – 1.04 | 1.44 ^***^ | 1.40 – 1.48 |
| Household size: 4 or more |  |  |  |  | 0.61 ^***^ | 0.59 – 0.62 | 1.28 ^***^ | 1.24 – 1.31 |
| No. of adults in employment in HH: 1 | Reference |  | Reference |  | Reference |  | Reference |  |
| No. of adults in employment in HH: 2 |  |  |  |  | 0.30 ^***^ | 0.30 – 0.30 | 0.61 ^***^ | 0.60 – 0.61 |
| No. of adults in employment in HH: 3 or more |  |  |  |  | 0.16 ^***^ | 0.16 – 0.16 | 0.43 ^***^ | 0.42 – 0.44 |
| Accommodation type: Detached whole house or bungalow | Reference |  | Reference |  | Reference |  | Reference |  |
| Accommodation type: Semi-detached whole house or bungalow |  |  |  |  | 0.97 ^***^ | 0.96 – 0.98 | 1.11 ^***^ | 1.10 – 1.12 |
| Accommodation type: Terraced whole house or bungalow |  |  |  |  | 0.89 ^***^ | 0.88 – 0.90 | 1.13 ^***^ | 1.12 – 1.15 |
| Accommodation type: Flat, maisonette or apartment |  |  |  |  | 0.85 ^***^ | 0.84 – 0.87 | 1.28 ^***^ | 1.25 – 1.30 |
| Accommodation type: Mobile or temporary structure |  |  |  |  | 0.96 | 0.88 – 1.04 | 1.13 ^**^ | 1.04 – 1.23 |
| Tenure of dwelling: Owned or shared ownership | Reference |  | Reference |  | Reference |  | Reference |  |
| Tenure of dwelling: Private rented |  |  |  |  | 0.49 ^***^ | 0.49 – 0.50 | 1.12 ^***^ | 1.10 – 1.14 |
| Tenure of dwelling: Social rented |  |  |  |  | 0.95 ^***^ | 0.94 – 0.97 | 1.44 ^***^ | 1.42 – 1.46 |
| Tenure of dwelling: Living rent free |  |  |  |  | 1.05 ^**^ | 1.01 – 1.09 | 1.21 ^***^ | 1.16 – 1.26 |
| NS-SEC of HH Reference Person: Higher managerial, administrative & professional occupations | Reference |  | Reference |  | Reference |  | Reference |  |
| NS-SEC of HH Reference Person: Intermediate occupations |  |  |  |  | 1.19 ^***^ | 1.17 – 1.21 | 1.07 ^***^ | 1.06 – 1.09 |
| NS-SEC of HH Reference Person: Lower supervisory & technical occupations |  |  |  |  | 1.23 ^***^ | 1.21 – 1.25 | 1.05 ^***^ | 1.03 – 1.07 |
| NS-SEC of HH Reference Person: Never worked & long-term unemployed |  |  |  |  | 0.68 ^***^ | 0.66 – 0.69 | 0.94 ^***^ | 0.92 – 0.97 |
| NS-SEC of HH Reference Person: Routine & Semi-routine occupations |  |  |  |  | 1.36 ^***^ | 1.35 – 1.38 | 1.07 ^***^ | 1.06 – 1.08 |
| NS-SEC of HH Reference Person: Small employers & own account workers |  |  |  |  | 1.27 ^***^ | 1.25 – 1.29 | 1.00 | 0.99 – 1.02 |
| Central heating: None | Reference |  | Reference |  | Reference |  | Reference |  |
| Central heating: Yes |  |  |  |  | 1.04 ^*^ | 1.01 – 1.07 | 1.20 ^***^ | 1.17 – 1.24 |
| Age grouping (10 yrs): 16 - 24 | Reference |  | Reference |  | Reference |  | Reference |  |
| Age grouping (10 yrs): 25 - 34 |  |  |  |  |  |  | 2.14 ^***^ | 2.09 – 2.20 |
| Age grouping (10 yrs): 35 - 44 |  |  |  |  |  |  | 4.28 ^***^ | 4.18 – 4.38 |
| Age grouping (10 yrs): 45 - 54 |  |  |  |  |  |  | 8.15 ^***^ | 7.96 – 8.34 |
| Age grouping (10 yrs): 55 - 64 |  |  |  |  |  |  | 16.12 ^***^ | 15.74 – 16.51 |
| Age grouping (10 yrs): 65 - 74 |  |  |  |  |  |  | 27.76 ^***^ | 27.06 – 28.47 |
| Age grouping (10 yrs): 75 - 84 |  |  |  |  |  |  | 61.10 ^***^ | 59.40 – 62.86 |
| RUC11Rural town & fringe |  |  | 1.11 ^***^ | 1.07 – 1.16 | 1.07 ^***^ | 1.04 – 1.11 | 1.04 | 1.00 – 1.07 |
| RUC11Rural village & dispersed |  |  | 1.06 ^*^ | 1.01 – 1.12 | 1.03 | 1.00 – 1.07 | 0.93 ^***^ | 0.90 – 0.97 |
| Age grouping (10 yrs): 85 & over |  |  |  |  |  |  | 79.46 ^***^ | 76.67 – 82.35 |
| Sex: Female | Reference |  | Reference |  | Reference |  | Reference |  |
| Sex: Male |  |  |  |  |  |  | 0.90 ^***^ | 0.89 – 0.90 |
| Ethnic group: White | Reference |  | Reference |  | Reference |  | Reference |  |
| Ethnic group: Mixed/multiple ethnic groups |  |  |  |  |  |  | 1.01 | 0.96 – 1.07 |
| Ethnic group: Asian/Asian British |  |  |  |  |  |  | 0.74 ^***^ | 0.72 – 0.77 |
| Ethnic group: Black/African/Caribbean/Black British |  |  |  |  |  |  | 0.61 ^***^ | 0.57 – 0.66 |
| Ethnic group: Other ethnic groups |  |  |  |  |  |  | 0.72 ^***^ | 0.66 – 0.78 |
| Provision of unpaid care (hrs): No | Reference |  | Reference |  | Reference |  | Reference |  |
| Provision of unpaid care (hrs): Yes |  |  |  |  |  |  | 0.93 ^***^ | 0.92 – 0.94 |
| Highest level of qualification: Level 4 | Reference |  | Reference |  | Reference |  | Reference |  |
| Highest level of qualification: Level 3 |  |  |  |  |  |  | 1.10 ^***^ | 1.08 – 1.12 |
| Highest level of qualification: Apprenticeship |  |  |  |  |  |  | 1.24 ^***^ | 1.21 – 1.27 |
| Highest level of qualification: Level 2 |  |  |  |  |  |  | 1.09 ^***^ | 1.08 – 1.11 |
| Highest level of qualification: Level 1 |  |  |  |  |  |  | 1.15 ^***^ | 1.13 – 1.16 |
| Highest level of qualification: Students |  |  |  |  |  |  | 0.37 ^***^ | 0.33 – 0.41 |
| Highest level of qualification: No qualifications |  |  |  |  |  |  | 1.48 ^***^ | 1.46 – 1.50 |
| Highest level of qualification: Others |  |  |  |  |  |  | 1.18 ^***^ | 1.16 – 1.21 |
| **Random Effects** | | | | | | | | |
| σ^2^ | 3.29 | | 3.29 | | 3.29 | | 3.29 | |
| τ_00_ | 0.82 _DWELLING_ID_PE:LSOA2011_CD_ | | 0.82 _DWELLING_ID_PE:LSOA2011_CD_ | | 0.23 _DWELLING_ID_PE:LSOA2011_CD_ | | 0.16 _DWELLING_ID_PE:LSOA2011_CD_ | |
|  | 0.13 _LSOA2011_CD_ | | 0.11 _LSOA2011_CD_ | | 0.06 _LSOA2011_CD_ | | 0.07 _LSOA2011_CD_ | |
| ICC | 0.22 | | 0.22 | | 0.08 | | 0.07 | |
| N | 948266 _DWELLING_ID_PE_ | | 948266 _DWELLING_ID_PE_ | | 948266 _DWELLING_ID_PE_ | | 948266 _DWELLING_ID_PE_ | |
|  | 1889 _LSOA2011_CD_ | | 1889 _LSOA2011_CD_ | | 1889 _LSOA2011_CD_ | | 1889 _LSOA2011_CD_ | |
| Observations | 1676432 | | 1676432 | | 1676432 | | 1676432 | |
| Marginal R^2^ / Conditional R^2^ | 0.000 / 0.224 | | 0.002 / 0.224 | | 0.247 / 0.308 | | 0.416 / 0.455 | |
| AIC | 2107215.007 | | 2107089.872 | | 1770324.158 | | 1543428.875 | |
| AICc | 2107215.007 | | 2107089.872 | | 1770324.159 | | 1543428.878 | |
| ** p<0.05   ** p<0.01   *** p<0.001* | | | | | | | | |

Table SM3-2 Fixed effects (Odds ratios) and random effects of MLMs of 3+ MM

| **Logit MLM of '3+ MLTC' in Wales: Fixed Effects for 3-Level Models** | | | | | | | | |
| --- | --- | --- | --- | --- | --- | --- | --- | --- |
|  | **Variance component** | | **Area effects** | | **Area & HH effects** | | **All Effects** | |
| *Predictors* | *Odds Ratios* | *CI* | *Odds Ratios* | *CI* | *Odds Ratios* | *CI* | *Odds Ratios* | *CI* |
| (Intercept) | 0.26 ^***^ | 0.26 – 0.27 | 0.22 ^***^ | 0.21 – 0.23 | 0.63 ^***^ | 0.60 – 0.67 | 0.01 ^***^ | 0.01 – 0.01 |
| WIMD2011 quintile: 5. Least | Reference |  | Reference |  | Reference |  | Reference |  |
| WIMD2011 quintile: 4 |  |  | 1.08 ^**^ | 1.02 – 1.14 | 1.04 | 1.00 – 1.08 | 1.06 ^**^ | 1.01 – 1.10 |
| WIMD2011 quintile: 3 |  |  | 1.15 ^***^ | 1.09 – 1.21 | 1.06 ^**^ | 1.02 – 1.10 | 1.10 ^***^ | 1.05 – 1.14 |
| WIMD2011 quintile: 2 |  |  | 1.24 ^***^ | 1.18 – 1.31 | 1.10 ^***^ | 1.06 – 1.14 | 1.16 ^***^ | 1.12 – 1.21 |
| WIMD2011 quintile: 1. Most |  |  | 1.39 ^***^ | 1.31 – 1.46 | 1.11 ^***^ | 1.07 – 1.16 | 1.28 ^***^ | 1.22 – 1.33 |
| No. of cars/vans in HH: No cars/vans | Reference |  | Reference |  | Reference |  | Reference |  |
| No. of cars/vans in HH: 1 car/van |  |  |  |  | 0.74 ^***^ | 0.74 – 0.75 | 0.90 ^***^ | 0.89 – 0.92 |
| No. of cars/vans in HH: 2 or more cars/vans |  |  |  |  | 0.64 ^***^ | 0.63 – 0.65 | 0.84 ^***^ | 0.82 – 0.85 |
| Family status: Couple family | Reference |  | Reference |  | Reference |  | Reference |  |
| Family status: Lone parent family |  |  |  |  | 0.51 ^***^ | 0.50 – 0.52 | 1.01 | 0.99 – 1.03 |
| Family status: Students, short-term migrants & others not in a family |  |  |  |  | 1.58 ^***^ | 1.54 – 1.62 | 1.31 ^***^ | 1.28 – 1.35 |
| Household size: 1 | Reference |  | Reference |  | Reference |  | Reference |  |
| Household size: 2 |  |  |  |  | 1.72 ^***^ | 1.68 – 1.77 | 1.50 ^***^ | 1.46 – 1.55 |
| Household size: 3 |  |  |  |  | 1.18 ^***^ | 1.15 – 1.22 | 1.69 ^***^ | 1.64 – 1.75 |
| Household size: 4 or more |  |  |  |  | 0.63 ^***^ | 0.62 – 0.65 | 1.49 ^***^ | 1.45 – 1.54 |
| No. of adults in employment in HH: 1 | Reference |  | Reference |  | Reference |  | Reference |  |
| No. of adults in employment in HH: 2 |  |  |  |  | 0.30 ^***^ | 0.30 – 0.30 | 0.62 ^***^ | 0.61 – 0.63 |
| No. of adults in employment in HH: 3 or more |  |  |  |  | 0.14 ^***^ | 0.14 – 0.14 | 0.39 ^***^ | 0.39 – 0.40 |
| Accommodation type: Detached whole house or bungalow | Reference |  | Reference |  | Reference |  | Reference |  |
| Accommodation type: Semi-detached whole house or bungalow |  |  |  |  | 1.01 | 1.00 – 1.02 | 1.13 ^***^ | 1.12 – 1.15 |
| Accommodation type: Terraced whole house or bungalow |  |  |  |  | 0.92 ^***^ | 0.91 – 0.93 | 1.14 ^***^ | 1.12 – 1.16 |
| Accommodation type: Flat, maisonette or apartment |  |  |  |  | 0.92 ^***^ | 0.90 – 0.94 | 1.31 ^***^ | 1.28 – 1.34 |
| Accommodation type: Mobile or temporary structure |  |  |  |  | 0.95 | 0.87 – 1.03 | 1.13 ^**^ | 1.03 – 1.24 |
| Tenure of dwelling: Owned or shared ownership | Reference |  | Reference |  | Reference |  | Reference |  |
| Tenure of dwelling: Private rented |  |  |  |  | 0.46 ^***^ | 0.45 – 0.47 | 1.18 ^***^ | 1.15 – 1.20 |
| Tenure of dwelling: Social rented |  |  |  |  | 0.96 ^***^ | 0.94 – 0.97 | 1.53 ^***^ | 1.50 – 1.55 |
| Tenure of dwelling: Living rent free |  |  |  |  | 1.18 ^***^ | 1.14 – 1.22 | 1.30 ^***^ | 1.25 – 1.36 |
| NS-SEC of HH Reference Person: Higher managerial, administrative & professional occupations | Reference |  | Reference |  | Reference |  | Reference |  |
| NS-SEC of HH Reference Person: Intermediate occupations |  |  |  |  | 1.19 ^***^ | 1.17 – 1.21 | 1.05 ^***^ | 1.03 – 1.07 |
| NS-SEC of HH Reference Person: Lower supervisory & technical occupations |  |  |  |  | 1.28 ^***^ | 1.26 – 1.30 | 1.06 ^***^ | 1.04 – 1.08 |
| NS-SEC of HH Reference Person: Never worked & long-term unemployed |  |  |  |  | 0.72 ^***^ | 0.71 – 0.74 | 0.99 | 0.97 – 1.02 |
| NS-SEC of HH Reference Person: Routine & Semi-routine occupations |  |  |  |  | 1.39 ^***^ | 1.38 – 1.41 | 1.08 ^***^ | 1.06 – 1.09 |
| NS-SEC of HH Reference Person: Small employers & own account workers |  |  |  |  | 1.34 ^***^ | 1.32 – 1.36 | 1.03 ^***^ | 1.02 – 1.05 |
| Central heating: None | Reference |  | Reference |  | Reference |  | Reference |  |
| Central heating: Yes |  |  |  |  | 1.05 ^**^ | 1.02 – 1.09 | 1.22 ^***^ | 1.18 – 1.27 |
| Age grouping (10 yrs): 16 - 24 | Reference |  | Reference |  | Reference |  | Reference |  |
| Age grouping (10 yrs): 25 - 34 |  |  |  |  |  |  | 2.82 ^***^ | 2.68 – 2.96 |
| Age grouping (10 yrs): 35 - 44 |  |  |  |  |  |  | 6.69 ^***^ | 6.39 – 6.99 |
| Age grouping (10 yrs): 45 - 54 |  |  |  |  |  |  | 14.57 ^***^ | 13.95 – 15.22 |
| Age grouping (10 yrs): 55 - 64 |  |  |  |  |  |  | 32.04 ^***^ | 30.67 – 33.48 |
| Age grouping (10 yrs): 65 - 74 |  |  |  |  |  |  | 55.61 ^***^ | 53.19 – 58.14 |
| Age grouping (10 yrs): 75 - 84 |  |  |  |  |  |  | 118.32 ^***^ | 113.06 – 123.84 |
| RUC11Rural town & fringe |  |  | 1.12 ^***^ | 1.07 – 1.17 | 1.08 ^***^ | 1.05 – 1.12 | 1.05 ^*^ | 1.01 – 1.09 |
| RUC11Rural village & dispersed |  |  | 1.05 | 1.00 – 1.11 | 1.05 ^**^ | 1.01 – 1.10 | 0.95 ^*^ | 0.91 – 0.99 |
| Age grouping (10 yrs): 85 & over |  |  |  |  |  |  | 171.19 ^***^ | 163.04 – 179.75 |
| Sex: Female | Reference |  | Reference |  | Reference |  | Reference |  |
| Sex: Male |  |  |  |  |  |  | 1.00 | 0.99 – 1.01 |
| Ethnic group: White | Reference |  | Reference |  | Reference |  | Reference |  |
| Ethnic group: Mixed/multiple ethnic groups |  |  |  |  |  |  | 1.03 | 0.97 – 1.11 |
| Ethnic group: Asian/Asian British |  |  |  |  |  |  | 0.82 ^***^ | 0.78 – 0.86 |
| Ethnic group: Black/African/Caribbean/Black British |  |  |  |  |  |  | 0.61 ^***^ | 0.55 – 0.67 |
| Ethnic group: Other ethnic groups |  |  |  |  |  |  | 0.75 ^***^ | 0.67 – 0.84 |
| Provision of unpaid care (hrs): No | Reference |  | Reference |  | Reference |  | Reference |  |
| Provision of unpaid care (hrs): Yes |  |  |  |  |  |  | 0.87 ^***^ | 0.86 – 0.88 |
| Highest level of qualification: Level 4 | Reference |  | Reference |  | Reference |  | Reference |  |
| Highest level of qualification: Level 3 |  |  |  |  |  |  | 1.07 ^***^ | 1.05 – 1.10 |
| Highest level of qualification: Apprenticeship |  |  |  |  |  |  | 1.25 ^***^ | 1.22 – 1.28 |
| Highest level of qualification: Level 2 |  |  |  |  |  |  | 1.08 ^***^ | 1.06 – 1.10 |
| Highest level of qualification: Level 1 |  |  |  |  |  |  | 1.15 ^***^ | 1.13 – 1.17 |
| Highest level of qualification: Students |  |  |  |  |  |  | 0.12 ^***^ | 0.10 – 0.15 |
| Highest level of qualification: No qualifications |  |  |  |  |  |  | 1.49 ^***^ | 1.47 – 1.51 |
| Highest level of qualification: Others |  |  |  |  |  |  | 1.23 ^***^ | 1.20 – 1.25 |
| **Random Effects** | | | | | | | | |
| σ^2^ | 3.29 | | 3.29 | | 3.29 | | 3.29 | |
| τ_00_ | 0.95 _DWELLING_ID_PE:LSOA2011_CD_ | | 0.95 _DWELLING_ID_PE:LSOA2011_CD_ | | 0.25 _DWELLING_ID_PE:LSOA2011_CD_ | | 0.18 _DWELLING_ID_PE:LSOA2011_CD_ | |
|  | 0.15 _LSOA2011_CD_ | | 0.13 _LSOA2011_CD_ | | 0.06 _LSOA2011_CD_ | | 0.07 _LSOA2011_CD_ | |
| ICC | 0.25 | | 0.25 | | 0.09 | | 0.07 | |
| N | 948266 _DWELLING_ID_PE_ | | 948266 _DWELLING_ID_PE_ | | 948266 _DWELLING_ID_PE_ | | 948266 _DWELLING_ID_PE_ | |
|  | 1889 _LSOA2011_CD_ | | 1889 _LSOA2011_CD_ | | 1889 _LSOA2011_CD_ | | 1889 _LSOA2011_CD_ | |
| Observations | 1676432 | | 1676432 | | 1676432 | | 1676432 | |
| Marginal R^2^ / Conditional R^2^ | 0.000 / 0.250 | | 0.003 / 0.249 | | 0.284 / 0.347 | | 0.487 / 0.524 | |
| AIC | 1715378.045 | | 1715207.370 | | 1405494.127 | | 1210995.870 | |
| AICc | 1715378.045 | | 1715207.370 | | 1405494.128 | | 1210995.873 | |
| ** p<0.05   ** p<0.01   *** p<0.001* | | | | | | | | |

Table SM3-3 Fixed effects (odds ratios) and random effects of MLMs of 3+ from 3+ MM

| **Logit MLM of '3+ MLTC from 3+ ICD-10 body systems' in Wales: Fixed Effects for 3-Level Models** | | | | | | | | |
| --- | --- | --- | --- | --- | --- | --- | --- | --- |
|  | **Variance component** | | **Area effects** | | **Area & HH effects** | | **All Effects** | |
| *Predictors* | *Odds Ratios* | *CI* | *Odds Ratios* | *CI* | *Odds Ratios* | *CI* | *Odds Ratios* | *CI* |
| (Intercept) | 0.19 ^***^ | 0.19 – 0.19 | 0.16 ^***^ | 0.16 – 0.17 | 0.40 ^***^ | 0.38 – 0.42 | 0.00 ^***^ | 0.00 – 0.00 |
| WIMD2011 quintile: 5. Least | Reference |  | Reference |  | Reference |  | Reference |  |
| WIMD2011 quintile: 4 |  |  | 1.07 ^*^ | 1.01 – 1.13 | 1.03 | 0.98 – 1.07 | 1.05 ^*^ | 1.00 – 1.10 |
| WIMD2011 quintile: 3 |  |  | 1.14 ^***^ | 1.07 – 1.20 | 1.05 ^*^ | 1.01 – 1.10 | 1.09 ^***^ | 1.04 – 1.14 |
| WIMD2011 quintile: 2 |  |  | 1.22 ^***^ | 1.16 – 1.29 | 1.08 ^***^ | 1.04 – 1.13 | 1.14 ^***^ | 1.09 – 1.19 |
| WIMD2011 quintile: 1. Most |  |  | 1.35 ^***^ | 1.28 – 1.43 | 1.10 ^***^ | 1.06 – 1.15 | 1.26 ^***^ | 1.21 – 1.32 |
| No. of cars/vans in HH: No cars/vans | Reference |  | Reference |  | Reference |  | Reference |  |
| No. of cars/vans in HH: 1 car/van |  |  |  |  | 0.75 ^***^ | 0.74 – 0.76 | 0.94 ^***^ | 0.93 – 0.96 |
| No. of cars/vans in HH: 2 or more cars/vans |  |  |  |  | 0.65 ^***^ | 0.64 – 0.66 | 0.87 ^***^ | 0.86 – 0.89 |
| Family status: Couple family | Reference |  | Reference |  | Reference |  | Reference |  |
| Family status: Lone parent family |  |  |  |  | 0.51 ^***^ | 0.50 – 0.52 | 1.01 | 0.99 – 1.03 |
| Family status: Students, short-term migrants & others not in a family |  |  |  |  | 1.59 ^***^ | 1.55 – 1.63 | 1.28 ^***^ | 1.25 – 1.32 |
| Household size: 1 | Reference |  | Reference |  | Reference |  | Reference |  |
| Household size: 2 |  |  |  |  | 1.72 ^***^ | 1.68 – 1.77 | 1.48 ^***^ | 1.43 – 1.52 |
| Household size: 3 |  |  |  |  | 1.20 ^***^ | 1.16 – 1.23 | 1.69 ^***^ | 1.64 – 1.75 |
| Household size: 4 or more |  |  |  |  | 0.63 ^***^ | 0.61 – 0.65 | 1.54 ^***^ | 1.49 – 1.60 |
| No. of adults in employment in HH: 1 | Reference |  | Reference |  | Reference |  | Reference |  |
| No. of adults in employment in HH: 2 |  |  |  |  | 0.32 ^***^ | 0.32 – 0.33 | 0.66 ^***^ | 0.66 – 0.67 |
| No. of adults in employment in HH: 3 or more |  |  |  |  | 0.15 ^***^ | 0.15 – 0.15 | 0.42 ^***^ | 0.42 – 0.43 |
| Accommodation type: Detached whole house or bungalow | Reference |  | Reference |  | Reference |  | Reference |  |
| Accommodation type: Semi-detached whole house or bungalow |  |  |  |  | 1.03 ^***^ | 1.02 – 1.04 | 1.14 ^***^ | 1.13 – 1.16 |
| Accommodation type: Terraced whole house or bungalow |  |  |  |  | 0.94 ^***^ | 0.93 – 0.96 | 1.16 ^***^ | 1.14 – 1.18 |
| Accommodation type: Flat, maisonette or apartment |  |  |  |  | 0.91 ^***^ | 0.89 – 0.93 | 1.24 ^***^ | 1.22 – 1.27 |
| Accommodation type: Mobile or temporary structure |  |  |  |  | 0.99 | 0.90 – 1.08 | 1.19 ^***^ | 1.09 – 1.31 |
| Tenure of dwelling: Owned or shared ownership | Reference |  | Reference |  | Reference |  | Reference |  |
| Tenure of dwelling: Private rented |  |  |  |  | 0.44 ^***^ | 0.43 – 0.45 | 1.15 ^***^ | 1.12 – 1.17 |
| Tenure of dwelling: Social rented |  |  |  |  | 0.94 ^***^ | 0.93 – 0.96 | 1.49 ^***^ | 1.46 – 1.51 |
| Tenure of dwelling: Living rent free |  |  |  |  | 1.20 ^***^ | 1.16 – 1.25 | 1.30 ^***^ | 1.24 – 1.35 |
| NS-SEC of HH Reference Person: Higher managerial, administrative & professional occupations | Reference |  | Reference |  | Reference |  | Reference |  |
| NS-SEC of HH Reference Person: Intermediate occupations |  |  |  |  | 1.19 ^***^ | 1.17 – 1.21 | 1.05 ^***^ | 1.03 – 1.07 |
| NS-SEC of HH Reference Person: Lower supervisory & technical occupations |  |  |  |  | 1.27 ^***^ | 1.25 – 1.30 | 1.06 ^***^ | 1.04 – 1.08 |
| NS-SEC of HH Reference Person: Never worked & long-term unemployed |  |  |  |  | 0.74 ^***^ | 0.72 – 0.76 | 0.99 | 0.96 – 1.02 |
| NS-SEC of HH Reference Person: Routine & Semi-routine occupations |  |  |  |  | 1.37 ^***^ | 1.35 – 1.39 | 1.07 ^***^ | 1.06 – 1.09 |
| NS-SEC of HH Reference Person: Small employers & own account workers |  |  |  |  | 1.32 ^***^ | 1.30 – 1.34 | 1.03 ^***^ | 1.02 – 1.05 |
| Central heating: None | Reference |  | Reference |  | Reference |  | Reference |  |
| Central heating: Yes |  |  |  |  | 1.08 ^***^ | 1.05 – 1.12 | 1.24 ^***^ | 1.19 – 1.28 |
| Age grouping (10 yrs): 16 - 24 | Reference |  | Reference |  | Reference |  | Reference |  |
| Age grouping (10 yrs): 25 - 34 |  |  |  |  |  |  | 2.76 ^***^ | 2.58 – 2.96 |
| Age grouping (10 yrs): 35 - 44 |  |  |  |  |  |  | 8.05 ^***^ | 7.56 – 8.57 |
| Age grouping (10 yrs): 45 - 54 |  |  |  |  |  |  | 20.36 ^***^ | 19.16 – 21.65 |
| Age grouping (10 yrs): 55 - 64 |  |  |  |  |  |  | 45.96 ^***^ | 43.23 – 48.86 |
| Age grouping (10 yrs): 65 - 74 |  |  |  |  |  |  | 78.92 ^***^ | 74.20 – 83.93 |
| Age grouping (10 yrs): 75 - 84 |  |  |  |  |  |  | 156.13 ^***^ | 146.70 – 166.16 |
| RUC11Rural town & fringe |  |  | 1.13 ^***^ | 1.07 – 1.18 | 1.09 ^***^ | 1.05 – 1.13 | 1.06 ^**^ | 1.02 – 1.10 |
| RUC11Rural village & dispersed |  |  | 1.04 | 0.99 – 1.10 | 1.05 ^*^ | 1.01 – 1.10 | 0.95 ^*^ | 0.91 – 0.99 |
| Age grouping (10 yrs): 85 & over |  |  |  |  |  |  | 217.21 ^***^ | 203.72 – 231.60 |
| Sex: Female | Reference |  | Reference |  | Reference |  | Reference |  |
| Sex: Male |  |  |  |  |  |  | 0.90 ^***^ | 0.89 – 0.91 |
| Ethnic group: White | Reference |  | Reference |  | Reference |  | Reference |  |
| Ethnic group: Mixed/multiple ethnic groups |  |  |  |  |  |  | 1.02 | 0.95 – 1.10 |
| Ethnic group: Asian/Asian British |  |  |  |  |  |  | 0.95 ^*^ | 0.90 – 1.00 |
| Ethnic group: Black/African/Caribbean/Black British |  |  |  |  |  |  | 0.71 ^***^ | 0.64 – 0.79 |
| Ethnic group: Other ethnic groups |  |  |  |  |  |  | 0.80 ^***^ | 0.71 – 0.90 |
| Provision of unpaid care (hrs): No | Reference |  | Reference |  | Reference |  | Reference |  |
| Provision of unpaid care (hrs): Yes |  |  |  |  |  |  | 0.88 ^***^ | 0.87 – 0.89 |
| Highest level of qualification: Level 4 | Reference |  | Reference |  | Reference |  | Reference |  |
| Highest level of qualification: Level 3 |  |  |  |  |  |  | 1.06 ^***^ | 1.04 – 1.09 |
| Highest level of qualification: Apprenticeship |  |  |  |  |  |  | 1.21 ^***^ | 1.18 – 1.24 |
| Highest level of qualification: Level 2 |  |  |  |  |  |  | 1.05 ^***^ | 1.03 – 1.07 |
| Highest level of qualification: Level 1 |  |  |  |  |  |  | 1.12 ^***^ | 1.10 – 1.15 |
| Highest level of qualification: Students |  |  |  |  |  |  | 0.13 ^***^ | 0.10 – 0.17 |
| Highest level of qualification: No qualifications |  |  |  |  |  |  | 1.43 ^***^ | 1.41 – 1.45 |
| Highest level of qualification: Others |  |  |  |  |  |  | 1.23 ^***^ | 1.20 – 1.26 |
| **Random Effects** | | | | | | | | |
| σ^2^ | 3.29 | | 3.29 | | 3.29 | | 3.29 | |
| τ_00_ | 0.85 _DWELLING_ID_PE:LSOA2011_CD_ | | 0.85 _DWELLING_ID_PE:LSOA2011_CD_ | | 0.24 _DWELLING_ID_PE:LSOA2011_CD_ | | 0.17 _DWELLING_ID_PE:LSOA2011_CD_ | |
|  | 0.15 _LSOA2011_CD_ | | 0.13 _LSOA2011_CD_ | | 0.07 _LSOA2011_CD_ | | 0.08 _LSOA2011_CD_ | |
| ICC | 0.23 | | 0.23 | | 0.09 | | 0.07 | |
| N | 948266 _DWELLING_ID_PE_ | | 948266 _DWELLING_ID_PE_ | | 948266 _DWELLING_ID_PE_ | | 948266 _DWELLING_ID_PE_ | |
|  | 1889 _LSOA2011_CD_ | | 1889 _LSOA2011_CD_ | | 1889 _LSOA2011_CD_ | | 1889 _LSOA2011_CD_ | |
| Observations | 1676432 | | 1676432 | | 1676432 | | 1676432 | |
| Marginal R^2^ / Conditional R^2^ | 0.000 / 0.233 | | 0.003 / 0.233 | | 0.275 / 0.339 | | 0.505 / 0.540 | |
| AIC | 1492722.249 | | 1492585.406 | | 1243213.175 | | 1077051.137 | |
| AICc | 1492722.249 | | 1492585.407 | | 1243213.176 | | 1077051.140 | |
| ** p<0.05   ** p<0.01   *** p<0.001* | | | | | | | | |

Table SM3-4 Fixed effects (Odds Ratios) and random effects of MLMs of mental-physical MM

| **Logit MLM of 'Mental-Physical MLTCs' in Wales: Fixed Effects for 3-Level Models** | | | | | | | | |
| --- | --- | --- | --- | --- | --- | --- | --- | --- |
|  | **Variance component** | | **Area effects** | | **Area & HH effects** | | **All Effects** | |
| *Predictors* | *Odds Ratios* | *CI* | *Odds Ratios* | *CI* | *Odds Ratios* | *CI* | *Odds Ratios* | *CI* |
| (Intercept) | 0.12 ^***^ | 0.12 – 0.12 | 0.10 ^***^ | 0.09 – 0.10 | 0.15 ^***^ | 0.14 – 0.16 | 0.02 ^***^ | 0.02 – 0.02 |
| ONS 2011 Rural-Urban class: Urban city & town | Reference |  | Reference |  | Reference |  | Reference |  |
| ONS 2011 Rural-Urban class: Rural town & fringe |  |  | 1.05 ^*^ | 1.01 – 1.09 | 1.04 ^*^ | 1.01 – 1.08 | 1.01 | 0.98 – 1.05 |
| ONS 2011 Rural-Urban class: Rural village & dispersed |  |  | 0.89 ^***^ | 0.85 – 0.92 | 0.95 ^**^ | 0.91 – 0.98 | 0.90 ^***^ | 0.87 – 0.94 |
| WIMD2011 quintile: 5. Least | Reference |  | Reference |  | Reference |  | Reference |  |
| WIMD2011 quintile: 4 |  |  | 1.11 ^***^ | 1.06 – 1.16 | 1.03 | 0.99 – 1.07 | 1.02 | 0.98 – 1.06 |
| WIMD2011 quintile: 3 |  |  | 1.23 ^***^ | 1.18 – 1.28 | 1.07 ^***^ | 1.03 – 1.11 | 1.06 ^**^ | 1.02 – 1.10 |
| WIMD2011 quintile: 2 |  |  | 1.42 ^***^ | 1.36 – 1.48 | 1.14 ^***^ | 1.09 – 1.18 | 1.12 ^***^ | 1.08 – 1.16 |
| WIMD2011 quintile: 1. Most |  |  | 1.74 ^***^ | 1.67 – 1.81 | 1.21 ^***^ | 1.16 – 1.25 | 1.20 ^***^ | 1.16 – 1.25 |
| No. of cars/vans in HH: No cars/vans | Reference |  | Reference |  | Reference |  | Reference |  |
| No. of cars/vans in HH: 1 car/van |  |  |  |  | 0.81 ^***^ | 0.79 – 0.82 | 0.85 ^***^ | 0.84 – 0.86 |
| No. of cars/vans in HH: 2 or more cars/vans |  |  |  |  | 0.75 ^***^ | 0.74 – 0.77 | 0.78 ^***^ | 0.76 – 0.79 |
| Family status: Couple family | Reference |  | Reference |  | Reference |  | Reference |  |
| Family status: Lone parent family |  |  |  |  | 0.83 ^***^ | 0.81 – 0.84 | 1.05 ^***^ | 1.03 – 1.07 |
| Family status: Students, short-term migrants & others not in a family |  |  |  |  | 1.34 ^***^ | 1.31 – 1.38 | 1.43 ^***^ | 1.39 – 1.48 |
| Household size: 1 | Reference |  | Reference |  | Reference |  | Reference |  |
| Household size: 2 |  |  |  |  | 1.24 ^***^ | 1.21 – 1.28 | 1.41 ^***^ | 1.37 – 1.46 |
| Household size: 3 |  |  |  |  | 1.14 ^***^ | 1.10 – 1.18 | 1.63 ^***^ | 1.58 – 1.69 |
| Household size: 4 or more |  |  |  |  | 0.83 ^***^ | 0.80 – 0.86 | 1.51 ^***^ | 1.46 – 1.56 |
| No. of adults in employment in HH: 1 | Reference |  | Reference |  | Reference |  | Reference |  |
| No. of adults in employment in HH: 2 |  |  |  |  | 0.54 ^***^ | 0.53 – 0.55 | 0.60 ^***^ | 0.59 – 0.61 |
| No. of adults in employment in HH: 3 or more |  |  |  |  | 0.31 ^***^ | 0.30 – 0.31 | 0.37 ^***^ | 0.36 – 0.37 |
| Accommodation type: Detached whole house or bungalow | Reference |  | Reference |  | Reference |  | Reference |  |
| Accommodation type: Semi-detached whole house or bungalow |  |  |  |  | 1.09 ^***^ | 1.07 – 1.10 | 1.12 ^***^ | 1.10 – 1.14 |
| Accommodation type: Terraced whole house or bungalow |  |  |  |  | 1.05 ^***^ | 1.03 – 1.06 | 1.12 ^***^ | 1.10 – 1.14 |
| Accommodation type: Flat, maisonette or apartment |  |  |  |  | 1.11 ^***^ | 1.08 – 1.13 | 1.31 ^***^ | 1.28 – 1.34 |
| Accommodation type: Mobile or temporary structure |  |  |  |  | 1.24 ^***^ | 1.12 – 1.37 | 1.27 ^***^ | 1.14 – 1.40 |
| Tenure of dwelling: Owned or shared ownership | Reference |  | Reference |  | Reference |  | Reference |  |
| Tenure of dwelling: Private rented |  |  |  |  | 0.87 ^***^ | 0.85 – 0.89 | 1.26 ^***^ | 1.23 – 1.28 |
| Tenure of dwelling: Social rented |  |  |  |  | 1.41 ^***^ | 1.38 – 1.43 | 1.52 ^***^ | 1.50 – 1.55 |
| Tenure of dwelling: Living rent free |  |  |  |  | 1.20 ^***^ | 1.15 – 1.25 | 1.30 ^***^ | 1.24 – 1.35 |
| NS-SEC of HH Reference Person: Higher managerial, administrative & professional occupations | Reference |  | Reference |  | Reference |  | Reference |  |
| NS-SEC of HH Reference Person: Intermediate occupations |  |  |  |  | 1.13 ^***^ | 1.11 – 1.15 | 1.04 ^***^ | 1.02 – 1.06 |
| NS-SEC of HH Reference Person: Lower supervisory & technical occupations |  |  |  |  | 1.09 ^***^ | 1.07 – 1.11 | 1.01 | 0.99 – 1.03 |
| NS-SEC of HH Reference Person: Never worked & long-term unemployed |  |  |  |  | 0.96 ^***^ | 0.93 – 0.98 | 1.00 | 0.97 – 1.02 |
| NS-SEC of HH Reference Person: Routine & Semi-routine occupations |  |  |  |  | 1.24 ^***^ | 1.22 – 1.26 | 1.06 ^***^ | 1.04 – 1.07 |
| NS-SEC of HH Reference Person: Small employers & own account workers |  |  |  |  | 1.13 ^***^ | 1.11 – 1.15 | 1.01 | 0.99 – 1.03 |
| Central heating: None | Reference |  | Reference |  | Reference |  | Reference |  |
| Central heating: Yes |  |  |  |  | 1.15 ^***^ | 1.11 – 1.20 | 1.20 ^***^ | 1.15 – 1.25 |
| Age grouping (10 yrs): 16 - 24 | Reference |  | Reference |  | Reference |  | Reference |  |
| Age grouping (10 yrs): 25 - 34 |  |  |  |  |  |  | 2.58 ^***^ | 2.48 – 2.68 |
| Age grouping (10 yrs): 35 - 44 |  |  |  |  |  |  | 5.30 ^***^ | 5.11 – 5.50 |
| Age grouping (10 yrs): 45 - 54 |  |  |  |  |  |  | 8.45 ^***^ | 8.16 – 8.76 |
| Age grouping (10 yrs): 55 - 64 |  |  |  |  |  |  | 10.06 ^***^ | 9.70 – 10.44 |
| Age grouping (10 yrs): 65 - 74 |  |  |  |  |  |  | 7.60 ^***^ | 7.31 – 7.89 |
| Age grouping (10 yrs): 75 - 84 |  |  |  |  |  |  | 7.49 ^***^ | 7.20 – 7.79 |
| Age grouping (10 yrs): 85 & over |  |  |  |  |  |  | 8.47 ^***^ | 8.12 – 8.84 |
| Sex: Female | Reference |  | Reference |  | Reference |  | Reference |  |
| Sex: Male |  |  |  |  |  |  | 0.66 ^***^ | 0.65 – 0.67 |
| Ethnic group: White | Reference |  | Reference |  | Reference |  | Reference |  |
| Ethnic group: Mixed/multiple ethnic groups |  |  |  |  |  |  | 1.01 | 0.94 – 1.08 |
| Ethnic group: Asian/Asian British |  |  |  |  |  |  | 0.45 ^***^ | 0.43 – 0.48 |
| Ethnic group: Black/African/Caribbean/Black British |  |  |  |  |  |  | 0.37 ^***^ | 0.33 – 0.42 |
| Ethnic group: Other ethnic groups |  |  |  |  |  |  | 0.59 ^***^ | 0.52 – 0.66 |
| Provision of unpaid care (hrs): No | Reference |  | Reference |  | Reference |  | Reference |  |
| Provision of unpaid care (hrs): Yes |  |  |  |  |  |  | 0.91 ^***^ | 0.90 – 0.93 |
| Highest level of qualification: Level 4 | Reference |  | Reference |  | Reference |  | Reference |  |
| Highest level of qualification: Level 3 |  |  |  |  |  |  | 1.08 ^***^ | 1.05 – 1.10 |
| Highest level of qualification: Apprenticeship |  |  |  |  |  |  | 1.10 ^***^ | 1.07 – 1.14 |
| Highest level of qualification: Level 2 |  |  |  |  |  |  | 1.11 ^***^ | 1.09 – 1.14 |
| Highest level of qualification: Level 1 |  |  |  |  |  |  | 1.18 ^***^ | 1.15 – 1.20 |
| Highest level of qualification: Students |  |  |  |  |  |  | 0.34 ^***^ | 0.29 – 0.41 |
| Highest level of qualification: No qualifications |  |  |  |  |  |  | 1.41 ^***^ | 1.38 – 1.43 |
| Highest level of qualification: Others |  |  |  |  |  |  | 1.19 ^***^ | 1.15 – 1.22 |
| **Random Effects** | | | | | | | | |
| σ^2^ | 3.29 | | 3.29 | | 3.29 | | 3.29 | |
| τ_00_ | 0.46 _DWELLING_ID_PE:LSOA2011_CD_ | | 0.46 _DWELLING_ID_PE:LSOA2011_CD_ | | 0.25 _DWELLING_ID_PE:LSOA2011_CD_ | | 0.29 _DWELLING_ID_PE:LSOA2011_CD_ | |
|  | 0.12 _LSOA2011_CD_ | | 0.07 _LSOA2011_CD_ | | 0.05 _LSOA2011_CD_ | | 0.05 _LSOA2011_CD_ | |
| ICC | 0.15 | | 0.14 | | 0.09 | | 0.09 | |
| N | 948266 _DWELLING_ID_PE_ | | 948266 _DWELLING_ID_PE_ | | 948266 _DWELLING_ID_PE_ | | 948266 _DWELLING_ID_PE_ | |
|  | 1889 _LSOA2011_CD_ | | 1889 _LSOA2011_CD_ | | 1889 _LSOA2011_CD_ | | 1889 _LSOA2011_CD_ | |
| Observations | 1676432 | | 1676432 | | 1676432 | | 1676432 | |
| Marginal R^2^ / Conditional R^2^ | 0.000 / 0.151 | | 0.011 / 0.148 | | 0.129 / 0.204 | | 0.252 / 0.323 | |
| AIC | 1162891.015 | | 1162124.341 | | 1085903.351 | | 1037858.226 | |
| AICc | 1162891.015 | | 1162124.342 | | 1085903.352 | | 1037858.229 | |
| ** p<0.05   ** p<0.01   *** p<0.001* | | | | | | | | |
