# Supplementary material for: Effects of household and neighbourhood attributes on four definitions of multimorbidity: a comparative multilevel analysis of linked clinical and census data of Wales
Source: BMJ Public Health. 2026 Jul 20;4(3):e002878. doi: 10.1136/bmjph-2025-002878 (PMC13386087; doi:10.1136/bmjph-2025-002878)
Supplement: online supplemental file 4 [file bmjph-4-3-s004.docx]

Effects of household and neighbourhood attributes on four definitions of multimorbidity: A comparative multilevel analysis of linked clinical and census data of Wales

Supplementary Material 4

# Level-wise decomposition of the ICC and R^2^ values of the models

The performance package in R ^1^, which implements methods by Johnson ^2^ and was used to compute both the R^2^ and ICC values as well as decompose them according to the grouping structure in the model. In effect, the neighbourhood ICC is equivalent to the level three R^2^, the household ICC is synonymous with the level two R^2^, while the level one R^2^ is equal to the conditional R^2^ minus the total ICC ^3^. These are shown in Table SM4-1 and Figure SM1 below.

Table SM4-1 R^2^ and ICC values of the models (with level-wise decomposition).

| **Definition of MM** | **Model specification** | **Marginal R^2^** | **Conditional R^2^** | **Total ICC** | **Household ICC** | **Neighbourhood ICC** |
| --- | --- | --- | --- | --- | --- | --- |
| 2+ MM | Model 1 | 0.0% | 22.4% | 22.4% | 19.4% | 3.0% |
| 2+ MM | Model 2 | 0.2% | 22.4% | 22.2% | 19.5% | 2.7% |
| 2+ MM | Model 3 | 24.7% | 30.8% | 8.1% | 6.4% | 1.7% |
| 2+ MM | Model 4 | 41.6% | 45.5% | 6.6% | 4.6% | 2.0% |
| 3+ MM | Model 1 | 0.0% | 25.0% | 25.0% | 21.6% | 3.3% |
| 3+ MM | Model 2 | 0.3% | 24.9% | 24.7% | 21.7% | 3.0% |
| 3+ MM | Model 3 | 28.4% | 34.7% | 8.8% | 7.0% | 1.8% |
| 3+ MM | Model 4 | 48.7% | 52.4% | 7.2% | 5.2% | 2.0% |
| 3+ LTC from 3+ Body Systems | Model 1 | 0.0% | 23.3% | 23.3% | 19.9% | 3.5% |
| 3+ LTC from 3+ Body Systems | Model 2 | 0.3% | 23.3% | 23.1% | 19.9% | 3.1% |
| 3+ LTC from 3+ Body Systems | Model 3 | 27.5% | 33.9% | 8.7% | 6.8% | 2.0% |
| 3+ LTC from 3+ Body Systems | Model 4 | 50.5% | 54.0% | 7.1% | 4.9% | 2.2% |
| Mental-physical MM | Model 1 | 0.0% | 15.1% | 15.1% | 11.9% | 3.2% |
| Mental-physical MM | Model 2 | 1.1% | 14.8% | 13.9% | 12.1% | 1.8% |
| Mental-physical MM | Model 3 | 12.9% | 20.4% | 8.6% | 7.0% | 1.5% |
| Mental-physical MM | Model 4 | 25.2% | 32.3% | 9.4% | 7.9% | 1.5% |


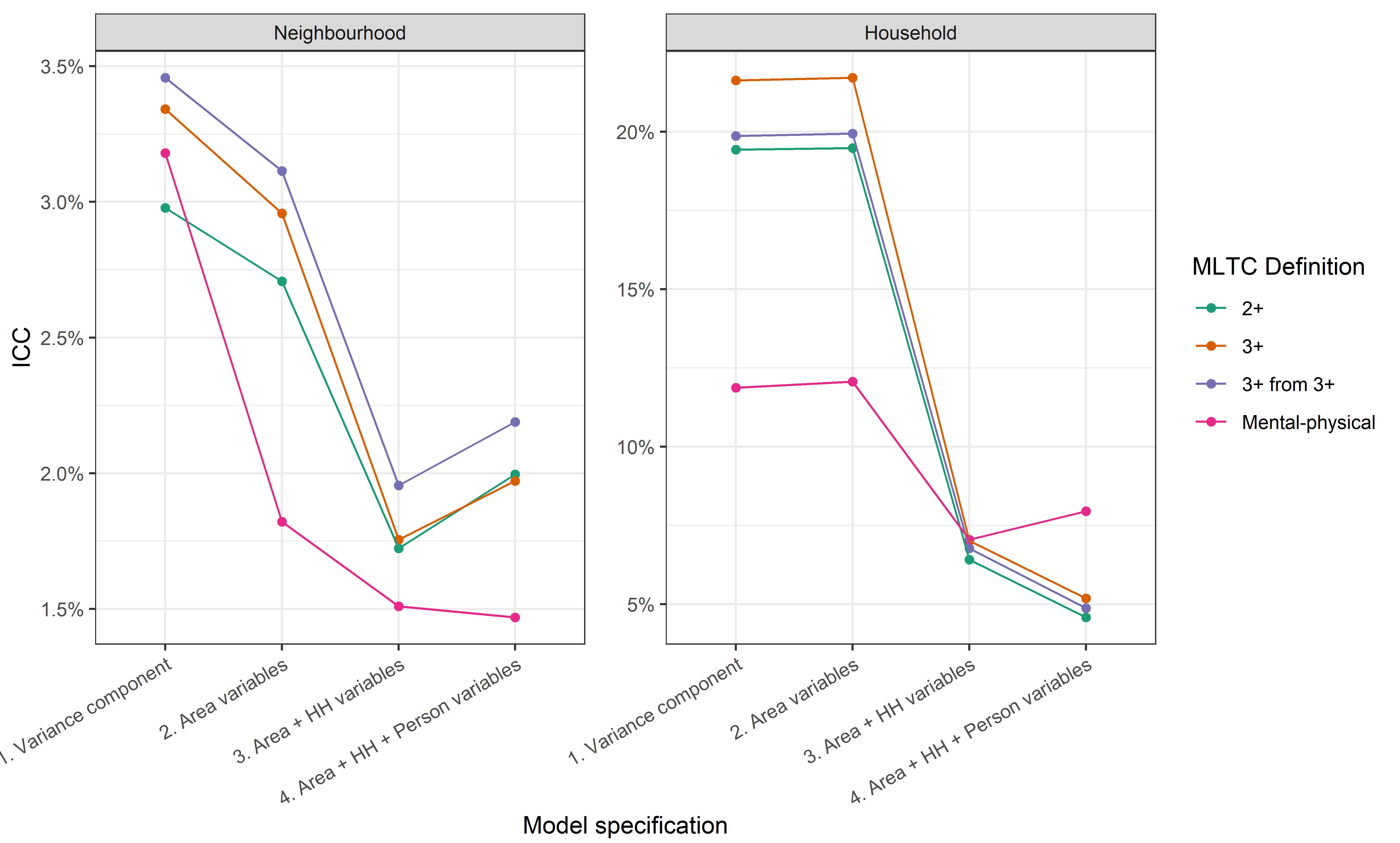


Figure SM4‑1 Dynamics of within-group clustering of multimorbidity risk in households and neighbourhoods

# References

1. Lüdecke D, Ben-Shachar MS, Patil I, et al. performance: An R package for assessment, comparison and testing of statistical models. *Journal of Open Source Software* 2021;6(60)

2. Johnson PCD. Extension of Nakagawa & Schielzeth's R2GLMM to random slopes models. *Methods in Ecology and Evolution* 2014;5(9):944-46. doi: <https://doi.org/10.1111/2041-210X.12225>

3. Nakagawa S, Johnson PCD, Schielzeth H. The coefficient of determination R2 and intra-class correlation coefficient from generalized linear mixed-effects models revisited and expanded. *Journal of The Royal Society Interface* 2017;14(134):20170213. doi: 10.1098/rsif.2017.0213
